# MicroRNAs and their regulatory networks in Chinese Gushi chicken abdominal adipose tissue during postnatal late development

**DOI:** 10.1186/s12864-019-6094-2

**Published:** 2019-10-25

**Authors:** Yi Chen, Yinli Zhao, Wenjiao Jin, Yuanfang Li, Yanhua Zhang, Xuejie Ma, Guirong Sun, Ruili Han, Yadong Tian, Hong Li, Xiangtao Kang, Guoxi Li

**Affiliations:** 1 College of Animal Science and Veterinary Medicine, Henan Agricultural University, Zheng zhou, Henan Province 450002 People’s Republic of China; 2 College of Biological Engineering, Henan University of Technology, Zheng zhou, Henan Province 450001 People’s Republic of China

**Keywords:** Abdominal adipose tissue, Chicken, Interaction networks, MicroRNA

## Abstract

**Background:**

Abdominal fat is the major adipose tissue in chickens. The growth status of abdominal fat during postnatal late development ultimately affects meat yield and quality in chickens. MicroRNAs (miRNAs) are endogenous small noncoding RNAs that regulate gene expression at the post-transcriptional level. Studies have shown that miRNAs play an important role in the biological processes involved in adipose tissue development. However, few studies have investigated miRNA expression profiles and their interaction networks associated with the postnatal late development of abdominal adipose tissue in chickens.

**Results:**

We constructed four small RNA libraries from abdominal adipose tissue obtained from Chinese domestic Gushi chickens at 6, 14, 22, and 30 weeks. A total of 507 known miRNAs and 53 novel miRNAs were identified based on the four small RNA libraries. Fifty-one significant differentially expressed (SDE) miRNAs were identified from six combinations by comparative analysis, and the expression patterns of these SDE miRNAs were divided into six subclusters by cluster analysis. Gene ontology enrichment analysis showed that the SDE miRNAs were primarily involved in the regulation of fat cell differentiation, regulation of lipid metabolism, regulation of fatty acid metabolism, and unsaturated fatty acid metabolism in the lipid metabolism- or deposition-related biological process categories. In addition, we constructed differentially expressed miRNA–mRNA interaction networks related to abdominal adipose development. The results showed that miRNA families, such as mir-30, mir-34, mir-199, mir-8, and mir-146, may have key roles in lipid metabolism, adipocyte proliferation and differentiation, and cell junctions during abdominal adipose tissue development in chickens.

**Conclusions:**

This study determined the dynamic miRNA transcriptome and characterized the miRNA–mRNA interaction networks in Gushi chicken abdominal adipose tissue for the first time. The results expanded the number of known miRNAs in abdominal adipose tissue and provide novel insights and a valuable resource to elucidate post-transcriptional regulation mechanisms during postnatal late development of abdominal adipose tissue in chicken.

## Background

The chicken is one of the most important agricultural animals [1]. Abdominal fat is the major adipose tissue in chickens. In poultry production, the characteristics of abdominal adipose tissue are closely related to the economic traits of chickens [2]. Excessive accumulation of abdominal fat often affects meat quality. Moreover, chickens can also be used as a biomedical model for studies on human metabolic disorders, such as insulin resistance, diabetes, obesity and metabolic syndrome [3, 4]. Therefore, understanding the molecular mechanisms of abdominal adipose tissue development can provide insight into biomedical research and benefit poultry genetic breeding and production in chickens. However, further research investigating the molecular mechanisms of chicken abdominal adipose tissue development is warranted.

The development of adipose tissue is a result of both adipocyte hyperplasia and hypertrophy. This complex process is regulated by a series of cellular and molecular events [5, 6]. MicroRNAs (miRNAs) are a class of ~ 22-nucleotide (nt)-long endogenous small noncoding RNAs that regulate gene expression at the post-transcriptional level in organisms; thus, they play an important role in many biological processes, including development, metabolism, and differentiation. A number of studies have shown that miRNAs also participate in the biological processes involved in adipose tissue development, including adipocyte proliferation and differentiation, adipogenesis, and lipid metabolism [7]. For example, miRNAs such as miR-143, miR-17–92 cluster, miR-21, miR-204, miR-378, and miR-375 have been shown to promote adipogenesis [8–13], while miRNAs such as miR-27b, let-7, miR-22, and miR-130 have been reported to impair adipocyte differentiation [14–17]. Therefore, miRNAs have become novel targets for investigating the molecular mechanisms related to adipose tissue development in animals [18–20]. Over the past decade, miRNAs in adipose tissues have been extensively studied. These studies have focused on the identification, expression, and function of miRNAs in adipose tissues of different anatomical depots [21–25], and these findings have helped to elucidate the molecular mechanisms underlying adipose tissue development in animals. However, previous studies were limited to humans, mice and a few agricultural animals, as well as metabolic diseases, such as obesity [26, 27].

Although 1232 miRNAs have been identified in the chicken genome (miRBase Version 22.1, October 2018), to date, there have been notably few reports on abdominal adipose tissue development in chickens [28]. In an earlier study, only 47 miRNAs were identified by molecular cloning experiments in abdominal adipose tissue from 28-day-old Arbor Acres commercial chickens [29]. More recently, Huang et al. (2015) identified 230 known miRNAs and 83 potentially novel miRNAs by deep sequencing in abdominal adipose tissues from a Chinese local breed and a commercial broiler line at 93 days of age and determined 62 differentially expressed miRNAs [30]. Two other studies detected 159 known miRNAs [31] and 33 differentially expressed miRNAs [32] in preadipocytes obtained from chicken abdominal adipose tissues, respectively. These studies helped us to understand miRNA functions in the development of chicken abdominal adipose tissues, but they also had the following limitations: i) they mainly concentrated on a few breeds and a specific stage of abdominal fat development; ii) they lacked an overall understanding of the regulatory mechanism of miRNAs during abdominal fat development in chickens as miRNAs function as an interactive network; and iii) they only identified a relatively small number of miRNAs using early experimental techniques. It is well-known that the physiological characteristics of abdominal adipose tissue are distinct between different stages of development in chickens post-hatching. The growth status of abdominal fat during postnatal late development ultimately affects meat yield and quality in chickens. However, there is still a paucity of knowledge on the regulatory mechanisms of miRNAs underlying abdominal adipose tissue development in the post-hatch late stage in different breeds. For these reasons, the identification of more miRNAs, construction of miRNA dynamic expression profiles, and further research investigating the interaction regulatory network of miRNAs are necessary to reveal the miRNA regulatory mechanism in chicken abdominal adipose tissue during postnatal late development.

The Gushi chicken is a domestic Chinese breed, and it is often used for breeding and production. To reveal the miRNA regulatory mechanism in the postnatal late development of abdominal fat in this variety, we constructed dynamic expression profiles of miRNAs in abdominal adipose tissue from Gushi chicken at 6, 14, 22, and 30 weeks after hatching. In addition, we also established the miRNA regulatory network involved in abdominal fat development based on the integrated analysis results of miRNA and mRNA expression profiles in Gushi chicken. The results expanded the number of known miRNAs in abdominal adipose tissue in chickens and provided a better understanding and novel insights into the miRNA regulatory mechanism of abdominal adipose tissue development in the postnatal late development stage of chickens.

## Results

### Small RNA library sequencing and sequence analysis

Using Illumina HiSeq2500 sequencing, we obtained 12,316,628, 26,800,794, 13,427,210 and 14,073,376 raw reads from the four small RNA libraries of Gushi chicken abdominal adipose tissue at 6 (z06), 14 (z14), 22 (z22), and 30 (z30) weeks old, respectively (Table 1). The raw sequence reads were deposited in the NCBI database Sequence Read Archive with the accession number PRJNA528858. After filtering, 11,220,507, 25,261,754, 12,928,203 and 13,520,406 high-quality clean reads measuring 18–35 nt were finally obtained from the z06, z14, z22 and z30 libraries, respectively. The lengths of these small RNAs were mainly 21–24 nt. In particular, the 22-nt small RNAs accounted for 47.35–58.54% of the total number of small RNAs in the libraries (Additional file 1: Figure S1). In addition, the 18–35-nt small RNA sequences were aligned with the chicken genome sequence. The results showed that 80.95, 85.63, 88.29 and 87.02% of the sequences from the z06, z14, z22 and z30 libraries were perfectly mapped to the chicken genome sequence, respectively (Table 1). These mapped sequences were used for subsequent miRNA identification.

**Table 1:** Statistics for the small RNA library sequences

Library	Raw reads	Clean reads			Mapped reads	
		Total reads	18-35 nt reads	18-35 nt uniq reads	Total reads	Uniq reads
z06	12,316,628	12,054,668 (97.87%)	11,220,507	1,593,408	9,083,101 (80.95%)	929,903
z14	26,800,794	26,324,597 (98.22%)	25,261,754	1,733,539	21,630,786 (85.63%)	1,075,445
z22	13,427,210	13,223,192 (98.48%)	12,928,203	557,408	11,414,249 (88.29%)	323,834
z30	14,073,376	13,838,865 (98.33%)	13,520,406	724,000	11,765,740 (87.02%)	449,550

The z06, z14, z22 and z30 represent the small RNA libraries obtained using abdominal adipose samples from Gushi chickens aged 6, 14, 22, and 30 weeks, respectively. The same abbreviations are used in the other tables

### MiRNAs expressed in Gushi chicken abdominal adipose tissues

By category annotation, the tRNA, rRNA, snoRNA, and other snRNA sequences in the clean reads were eliminated (Additional file 7: Table S1). The remaining sequences were then compared to the mature sequences of miRNAs from chickens in miRBase (Release 22.0). The 11,460 unique sequences (24,059,167 reads) were fully matched to the miRNAs of the chicken. Combining structural predictions of precursor sequences, a total of 507 known miRNAs and 53 novel miRNAs were identified from the four small RNA libraries in Gushi chicken abdominal adipose tissue (Additional file 11). Detailed information on novel miRNAs is shown in Additional file 8: Table S2. The statistics for the miRNAs identified in each library are shown in Table 2.

**Table 2:** Statistics for the miRNAs identified in each library

Types	Total	z06	z14	z22	z30
Known miRNA					
Mapped mature	507	382	414	363	376
Mapped hairpin	428	324	356	296	314
Mapped uniq sRNA	11,460	2613	3366	2620	2861
Mapped total sRNA	24,059,167	3,722,661	9,513,607	5,259,223	5,563,676
Novel miRNA					
Mapped mature	53	34	40	27	33
Mapped hairpin	56	37	45	30	38
Mapped uniq sRNA	326	75	109	61	81
Mapped total sRNA	6954	1707	2567	1376	1304

**Fig. 1:**
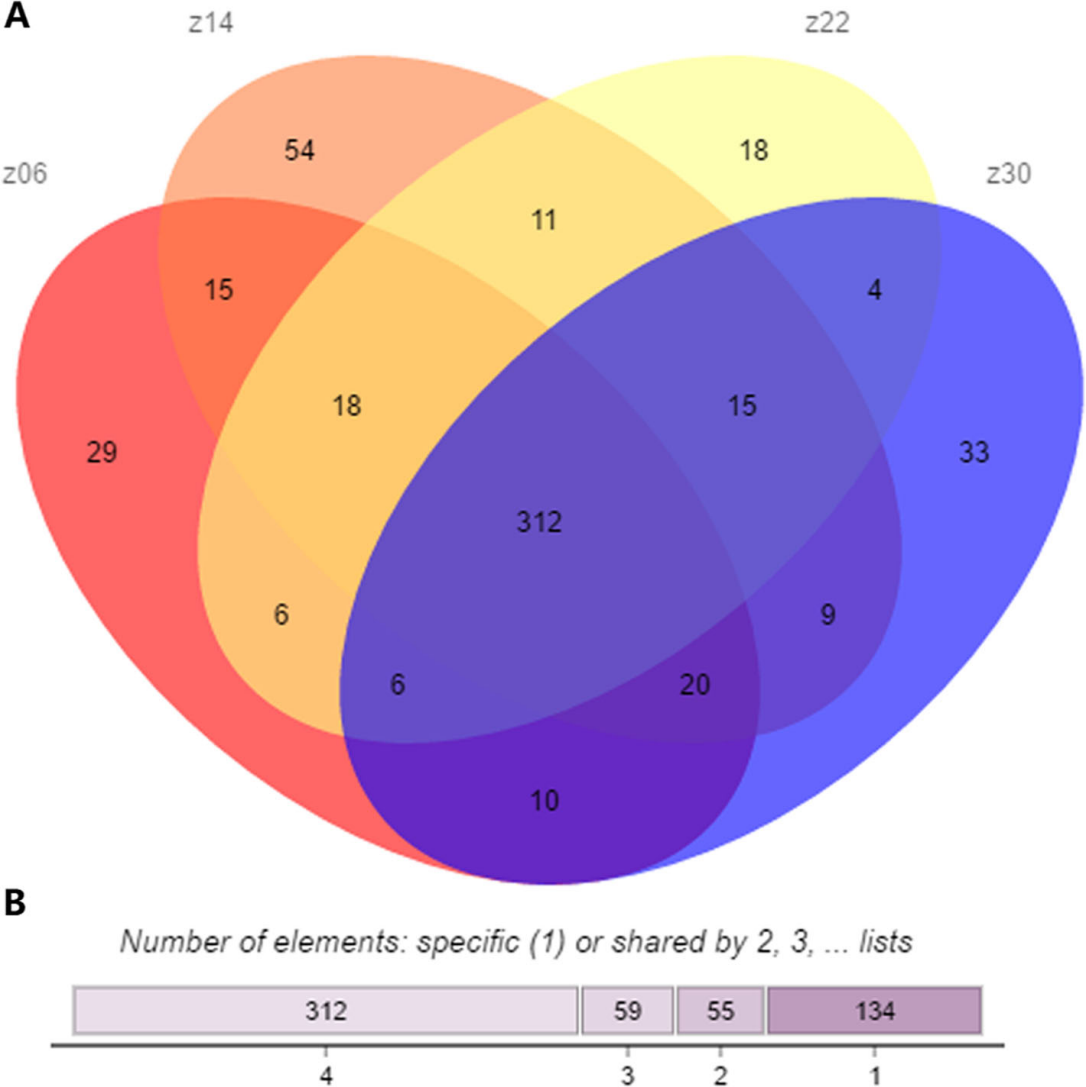
Venn diagram (**a**) and shared case (**b**) of miRNAs in the four developmental stages of Gushi chicken abdominal adipose tissue. The z06, z14, z22 and z30 represent the small RNA libraries obtained using abdominal adipose samples from Gushi chickens aged 6, 14, 22, and 30 weeks, respectively

In addition, we also analysed the expression levels of 560 identified miRNAs in different developmental stages of Gushi chicken abdominal adipose tissue. Among these miRNAs, 312 miRNAs were expressed in all four developmental stages, while 29, 54, 18, and 33 miRNAs were expressed specifically at 6, 14, 22, and 30 weeks of age, respectively (Fig. 1 and Additional file 11). This finding indicated that there were differences in the miRNA expression profiles during different developmental stages of chicken abdominal fat. In addition, the abundance of these identified microRNAs was also different. Most of the novel miRNAs were relatively weakly expressed in the abdominal adipose tissue samples, while the abundance of miRNAs expressed specifically in different developmental stages of abdominal adipose tissue was also considerably lower (Additional file 11). The percentages of miRNAs with TPM > 60 in z06, z14, z22, and z30 were 18.38, 19.29, 19.64, and 18.57%, respectively, which suggests that only a few miRNAs were abundantly expressed during abdominal adipose tissue development in chicken. Of these abundant miRNAs, some dominated the miRNA libraries in different developmental stages of abdominal fat. For example, the expression level of miR-148a-3p was the highest in all four developmental stages, and its reads in the z06, z14, z22, and z30 libraries accounted for 7.53, 12.95, 14.08, and 9.95% of total clean reads, respectively.

In addition, family analysis of the identified miRNAs was also carried out. The results showed that 207 miRNA precursors belonged to 113 miRNA gene families, which accounted for 42.77% of the identified precursors (Additional file 12). Among these miRNA families, 45 families contained more than two precursors, and most members of these families were expressed in higher abundance in chicken abdominal adipose tissue. In particular, nine members of the let-7 miRNA family were detected in chicken abdominal adipose tissue. The miRNA families mir-30, mir-15, mir-17, mir-10, mir-196, and mir-130 also had seven, six, six, five, five, and five miRNA precursors, respectively. In addition, the conservation analysis showed that 24 miRNA families were present both in invertebrates and vertebrates. Twenty-three miRNA families existed only in chickens, including mir-3607, mir-6608, mir-1781, mir-2131, mir-1729, mir-1744, mir-1451, mir-1434, mir-1684, mir-1467, mir-1460, mir-1803, mir-6648, mir-3064, mir-1641, mir-1634, mir-7458, mir-1698, mir-1559, mir-1784, mir-2954, mir-1756, and mir-3524 (Additional file 12). These results indicated that there were many highly conserved miRNAs and specific miRNAs during abdominal adipose tissue development in chickens.

### Significant differentially expressed (SDE) miRNAs during abdominal adipose tissue development

**Fig. 2:**
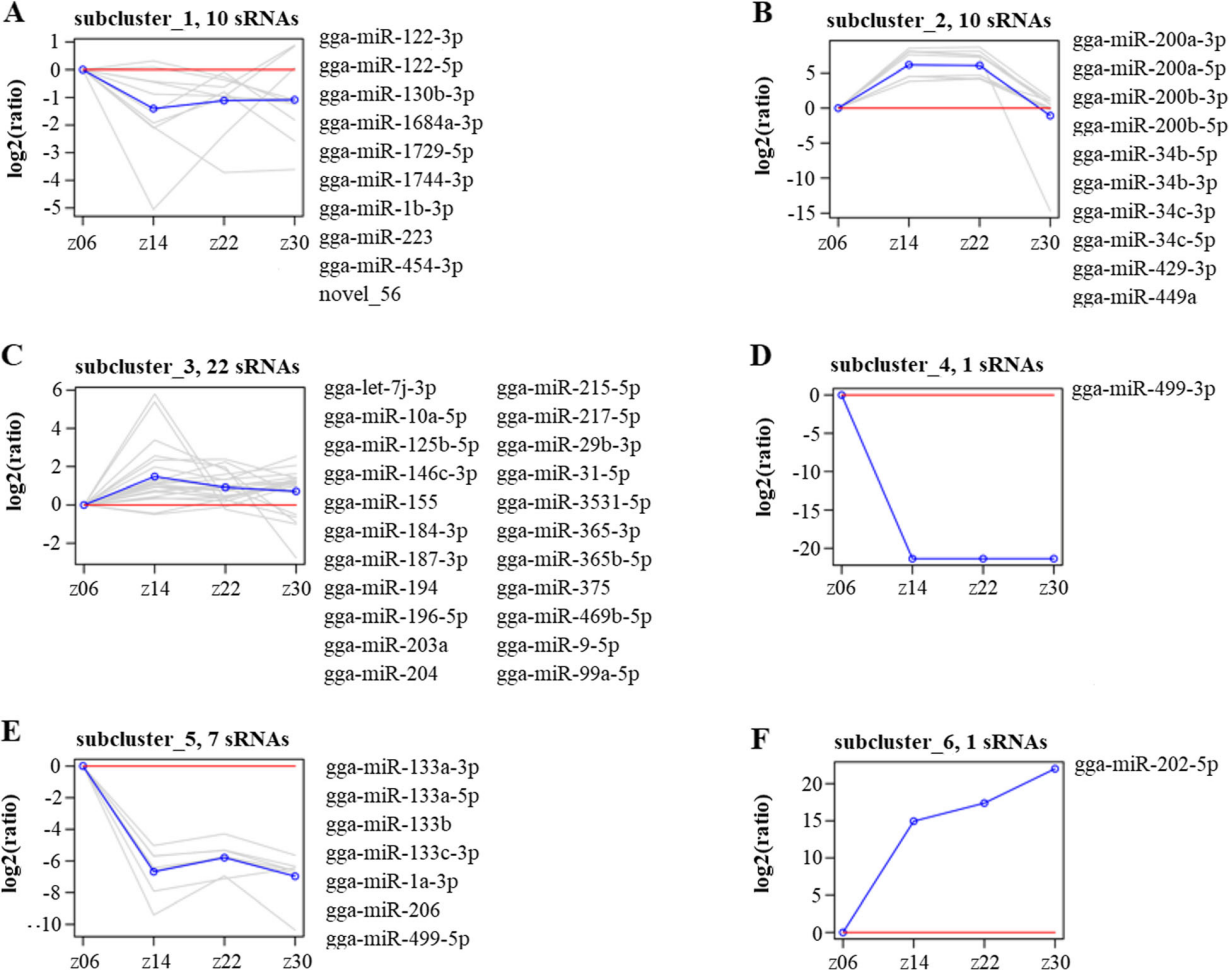
K-means clustering of differentially expressed miRNAs. (**a**), (**b**), (**c**), (**d**), (**e**) and (**f**) represent different modes of expression, respectively The gray line in each graph is a line chart representing the relative expression level of a given miRNA in the cluster for the four developmental stages of abdominal adipose tissue. The blue line in each graph is a line chart representing the relative mean expression level of all miRNAs of the cluster for the four developmental stages of abdominal fat. The red line is for reference where miRNAs above the red line were upregulated and those below the red line were downregulated. The x-axis indicates the four developmental stages of abdominal fat and the y-axis indicates the relative expression levels of miRNAs

**Fig. 3:**
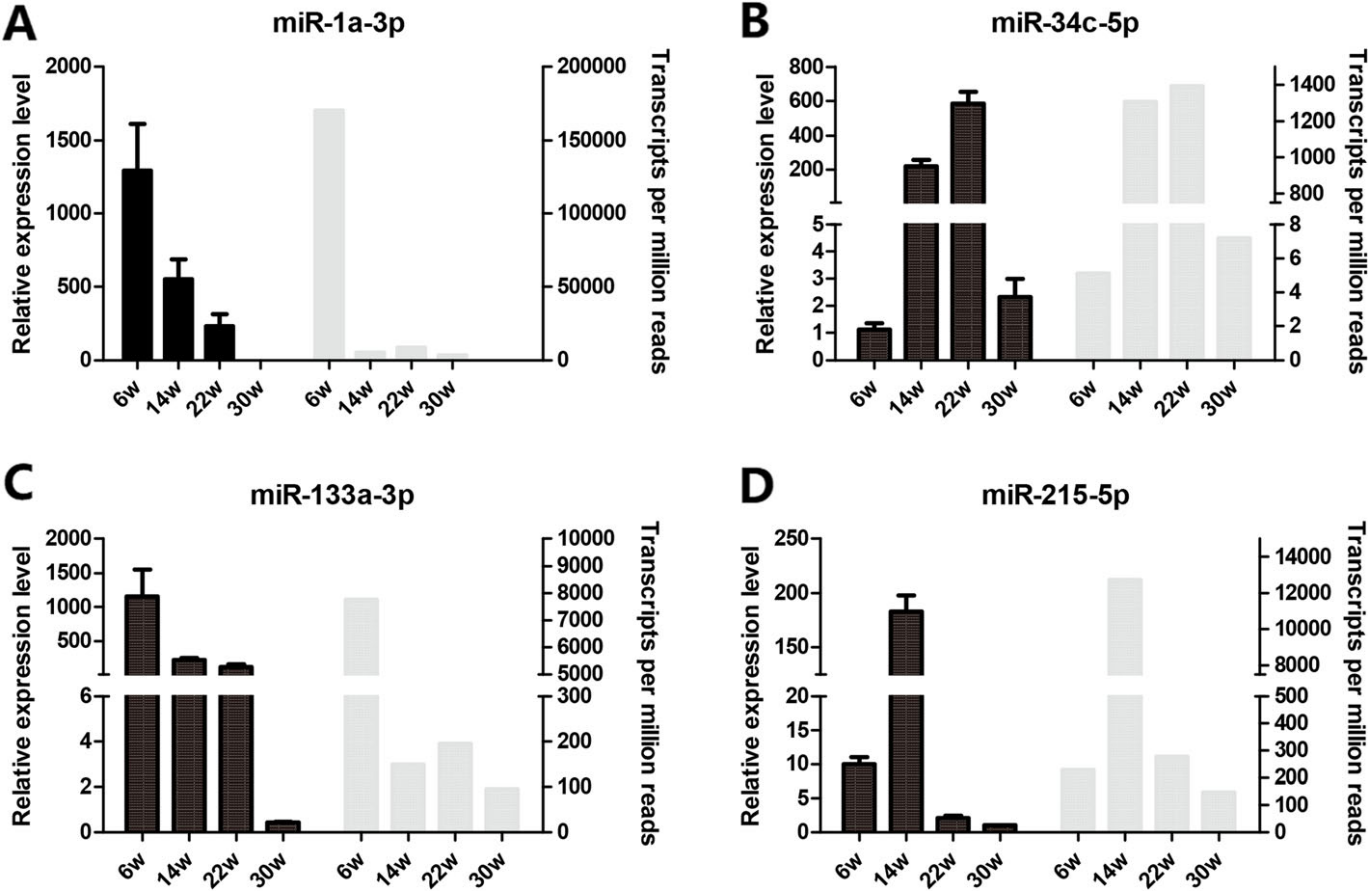
The qRT-PCR verification of SDE miRNAs. (**a**), (**b**), (**c**) and (**d**) The qRT-PCR verification of miR-1a-3p, miR-34c-5p, miR-133a-3p, miR-215b-5p, respectively. The 6w, 14w, 22w, and 30w represent the samples obtained at 6, 14, 22, and 30 weeks, respectively. Data are represented by the mean ± SEM (*n* = 3) in qRT-PCR results

The expression levels of the identified miRNAs in Gushi chicken abdominal fat at 6, 14, 22, and 30 weeks were compared. Under the criteria of a q value < 0.01 and | log_2_ (fold change) | > 1, a total of 51 SDE miRNAs were determined from the z14 vs. z06, z22 vs. z06, z30 vs. z06, z22 vs. z14, z30 vs. z14, and z30 vs. z22 combination (Additional file 9: Table S3). In particular, there were 34 SDE miRNAs (12 downregulated and 22 upregulated) in z14 vs. z06, and there were 24 SDE miRNAs (19 downregulated and 5 upregulated) in z30 vs. z22 (Table 3), and these two combinations had more SDE miRNAs than the other combinations. This finding suggested that 6–14 weeks and 22–30 weeks were important stages in the development and deposition of abdominal adipose tissue in Gushi chicken. Of these SDE miRNAs, gga-miR-215-5p, gga-miR-122-5p and gga-miR-499-5p were expressed differently in four developmental stages of Gushi chicken abdominal adipose tissue. The above 51 SDE miRNAs were clustered into six subclusters (Fig. 2). Thus, the expression level of SDE miRNAs, such as gga-miR-122-3p (Fig. 2a), gga-miR-499-3p (Fig. 2d), and gga-miR-133a-3p (Fig. 2e), decreased gradually with the development of abdominal fat. In contrast, the expression levels of gga-miR-202-5p increased with the development of abdominal adipose tissue (Fig. 2f), while SDE miRNAs, such as gga-miR-200a-3p, were expressed in high abundance between 14 weeks and 22 weeks (Fig. 2b). The above results indicated that these SDE miRNAs might be closely related to the development of chicken abdominal fat. Finally, miR-215-5p, miR-133a-3p, miR-34c-5p and miR-1a-3p were randomly selected for qRT-PCR validation. Their expression levels determined by qRT-PCR showed similar patterns in comparison to the RNA-seq data (Fig. 3), which indicated that the sequencing analysis results were reliable in this study.

**Table 3:** Details of the partial SDE miRNAs identified in this study

miRNA ID	z14 vs z06			z22 vs z14			z30 vs z22		
	log2(FC)	*q*-value	Signifi	log2(FC)	*q*-value	Signifi	log2(FC)	*q*-value	Signifi
gga-miR-34b-5p	8.742	0	Yes	0.069	3.57 E-04	No	−7.481	0	Yes
gga-miR-34c-3p	8.346	6.86E-32	Yes	−0.696	7.87 E-03	No	−6.706	1.12E-23	Yes
gga-miR-34b-3p	8.244	2.15E-88	Yes	− 0.669	1.47E-06	No	−7.101	1.25E-64	Yes
gga-miR-34c-5p	8.223	3.02E-274	Yes	0.002	4.01 E-01	No	−7.793	6.88E-303	Yes
gga-miR-449a	7.820	4.22E-12	Yes	−0.452	7.66 E-01	No	−6.434	3.09E-10	Yes
gga-miR-215-5p	6.026	0	Yes	−5.611	0	Yes	−1.116	9.90E-10	Yes
gga-miR-194	5.611	1.38E-296	Yes	−5.709	3.17E-296	Yes	−0.959	2.82 E-01	No
gga-miR-200b-3p	4.769	0	Yes	−0.446	5.68E-15	No	−3.363	0	Yes
gga-miR-200a-5p	4.755	6.31E-19	Yes	−0.426	5.82 E-01	No	−4.279	2.38E-14	Yes
gga-miR-200a-3p	4.727	0	Yes	0.136	5.62E-08	No	−5.046	0	Yes
gga-miR-429-3p	4.042	2.54E-26	Yes	0.258	2.05 E-01	No	−4.595	5.92E-36	Yes
gga-miR-200b-5p	4.030	1.51 E-04	Yes	0.409	7.85 E-01	No	−4.819	9.93E-07	Yes
gga-miR-217-5p	3.607	6.05E-06	Yes	−1.652	1.44 E-02	No	−2.918	4.01 E-02	Yes
gga-miR-375	2.780	2.54E-26	Yes	−0.680	3.46 E-03	No	−4.930	5.08E-28	Yes
gga-miR-31-5p	2.561	3.87 E-03	Yes	−0.049	9.99 E-01	No	−1.180	2.33 E-01	No
gga-miR-187-3p	2.201	8.48 E-04	Yes	−0.775	4.29 E-01	No	1.044	5.76 E-03	Yes
gga-miR-203a	1.546	1.14 E-04	Yes	−0.478	5.26 E-01	No	−1.639	3.85 E-04	Yes
gga-miR-9-5p	1.308	2.54E-11	Yes	0.152	2.42 E-01	No	0.093	4.22 E-02	No
gga-miR-204	1.280	2.29 E-04	Yes	−0.365	7.66 E-01	No	0.268	1.20 E-01	No
gga-miR-155	1.174	1.42E-06	Yes	−0.551	4.04 E-02	No	0.293	1.52 E-02	No
gga-miR-99a-5p	1.168	0	Yes	−0.299	1.65E-72	No	−0.157	8.36 E-03	No
gga-miR-10a-5p	1.148	0	Yes	−0.065	4.80 E-02	No	0.285	0	No
gga-miR-499-5p	−9.183	0	Yes	2.367	6.87E-21	Yes	−3.607	4.78E-29	Yes
gga-miR-206	−7.695	1.48E-129	Yes	0.702	9.99 E-01	No	0.487	8.43 E-01	No
gga-miR-133a-5p	−6.395	3.78E-70	Yes	0.813	9.72 E-01	No	−1.094	7.66 E-01	No
gga-miR-133b	−6.207	8.52E-75	Yes	0.541	9.99 E-01	No	−1.181	7.53 E-01	No
gga-miR-499-3p	−5.634	4.45E-07	Yes	–	–	–	–	–	–
gga-miR-133a-3p	−5.484	0	Yes	0.299	7.40 E-02	No	−1.225	2.42E-08	Yes
gga-miR-133c-3p	−5.459	1.08E-248	Yes	0.244	9.99 E-01	No	−1.479	4.01 E-02	No
gga-miR-1744-3p	−4.831	8.87E-05	Yes	2.612	4.80 E-01	No	2.227	4.06 E-03	Yes
gga-miR-1a-3p	−4.810	0	Yes	0.655	4.58E-197	No	−1.550	0	Yes
gga-miR-1b-3p	−1.848	4.41E-122	Yes	−1.739	1.33E-21	Yes	−0.082	9.90 E-01	No
gga-miR-1729-5p	−1.703	4.82E-13	Yes	0.819	2.04 E-02	No	−0.243	9.94 E-01	No
gga-miR-122-5p	−1.316	3.46E-237	Yes	1.368	1.95E-206	Yes	−1.929	3.72E-266	Yes
gga-miR-202-5p	–	–	–	2.349	8.94 E-01	No	4.431	2.93E-10	Yes
gga-miR-1684a-3p	−0.221	7.36 E-01	No	−0.619	9.45 E-01	No	1.623	2.49 E-04	Yes
gga-miR-223	−0.1997	9.72 E-02	No	−0.291	9.45 E-01	No	1.305	7.01E-18	Yes
gga-miR-460b-5p	1.453	2.75 E-01	No	0.920	2.04 E-01	No	−1.906	9.18 E-03	Yes
gga-miR-454-3p	0.535	2.58 E-01	No	−0.664	1.71 E-02	No	−1.185	3.73 E-04	Yes

### Target gene predictions and functional analysis

**Fig. 4:**
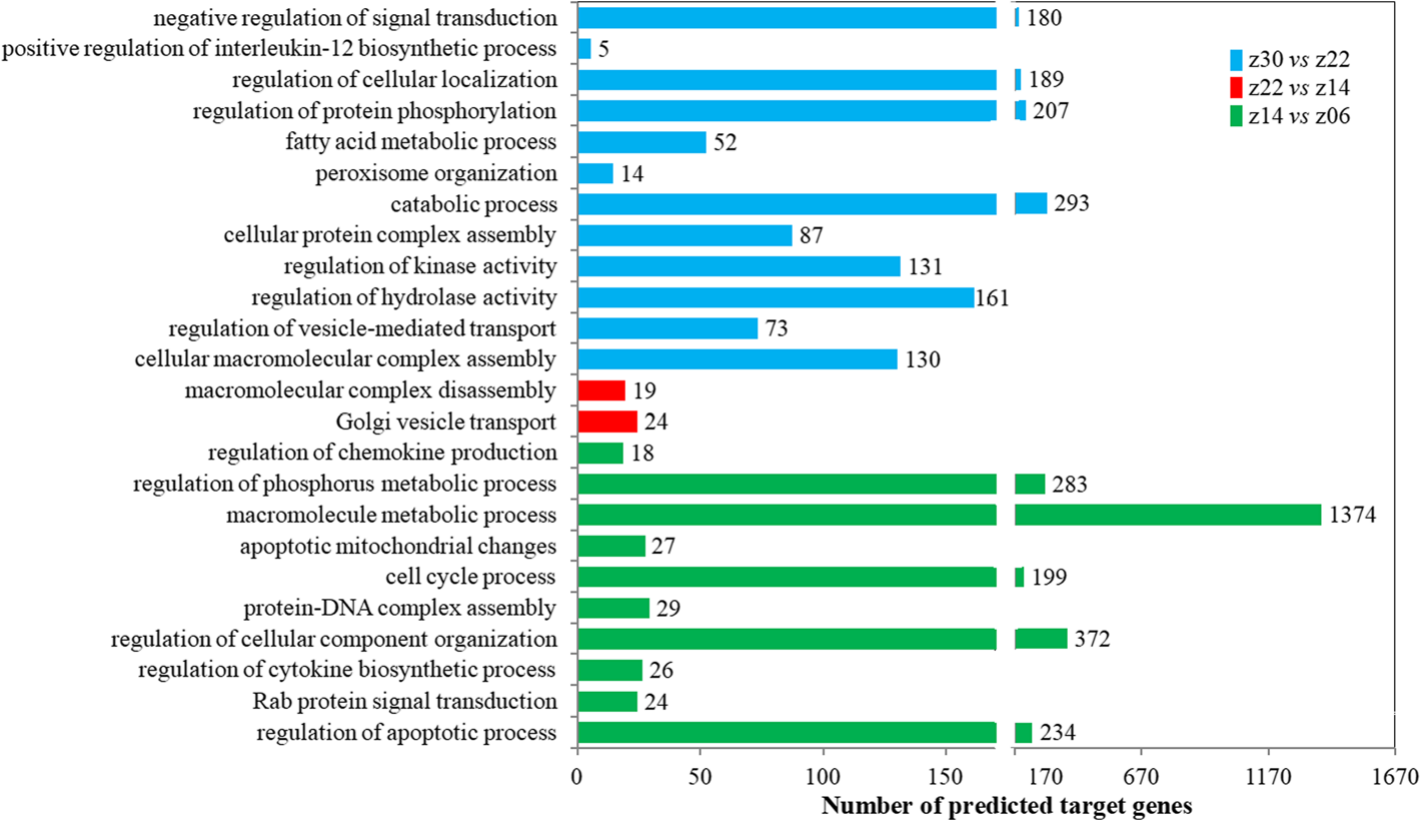
Comparison of different biological processes associated to the SDE miRNAs in the z14 vs. z06, z22 vs. z14 and z30 vs. z22 combinations. The y-axis indicates the significantly enriched GO terms associated to the SDE miRNAs in the different comparisons, and the x-axis indicates the number of predicted target genes enriched in the corresponding GO terms. The number of background genes was 13,601

Based on the SDE miRNAs identified in different combinations, 3489, 3212, 3071, 1105, 3067 and 3113 target genes were predicted in the z14 vs. z06, z22 vs. z06, z30 vs. z06, z22 vs. z14, z30 vs. z14, and z30 vs. z22 combination, respectively. Therefore, 843 predicted target genes were shared among all six combinations, while the number of unique target genes in the z14 vs. z06, Z22 vs. z06, Z30 vs. z06, Z22 vs. z14 and Z30 vs. z22 combination were 21, 12, 110, 17 and 43, respectively (Additional file 2: Figure S2). Subsequently, the predicted target genes of SDE miRNAs were analysed by GO enrichment. In the biological process category, 108, 106, 74, 8, 86 and 107 significantly enriched GO terms were obtained from the z14 vs. z06, Z22 vs. z06, Z30 vs. z06, Z22 vs. z14, Z30 vs. z14 and Z30 vs. z22 combination, respectively. Most of these GO terms did not differ significantly between combinations. Their functions were mainly concentrated in the biological process related to localization, metabolic processes and transport (Additional file 3: Figure S3), for instance, cellular macromolecule localization, cellular metabolic processes, cellular protein localization, cytoplasmic transport, intracellular transport, macromolecule localization, and primary metabolic processes. However, the SDE miRNA biological processes were also different in the different developmental stages of Gushi chicken abdominal adipose tissue. The significantly enriched GO terms in the z14 vs. z06 combination were mainly involved in the cell cycle process, regulation of the apoptotic process, and regulation of cellular component organization, and the GO terms in the z22 vs. z14 combination were mainly related to Golgi vesicle transport and macromolecular complex disassembly, while the GO terms in the z30 vs. z22 combination were mainly focused on cellular protein complex assembly, fatty acid metabolism, and regulation of kinase activity (Fig. 4 and Additional file 13). Moreover, we also found many GO terms related to lipid metabolism or fat deposition in the GO enrichment results, although these GO terms were not significantly enriched within combinations. In particular, some important biological processes involved in the regulation of fat cell differentiation, lipid metabolism, fatty acid metabolism and unsaturated fatty acid metabolism were the same in the three combinations, z14 vs. z06, z22 vs. z14 and z30 vs. z22 (Additional file 10: Table S4). Only a few biological processes were different, including acylglycerol homeostasis, medium-chain fatty acid metabolism, and lipid hydroperoxide transport (Additional file 4: Figure S4). These results suggested that the basic biological processes in which SDE miRNAs participated were similar in the development of Gushi chicken abdominal adipose tissue from 6 weeks to 30 weeks, but there were differences in key biological processes at different developmental stages.

### Interaction networks of miRNAs during abdominal adipose tissue development

**Fig. 5:**
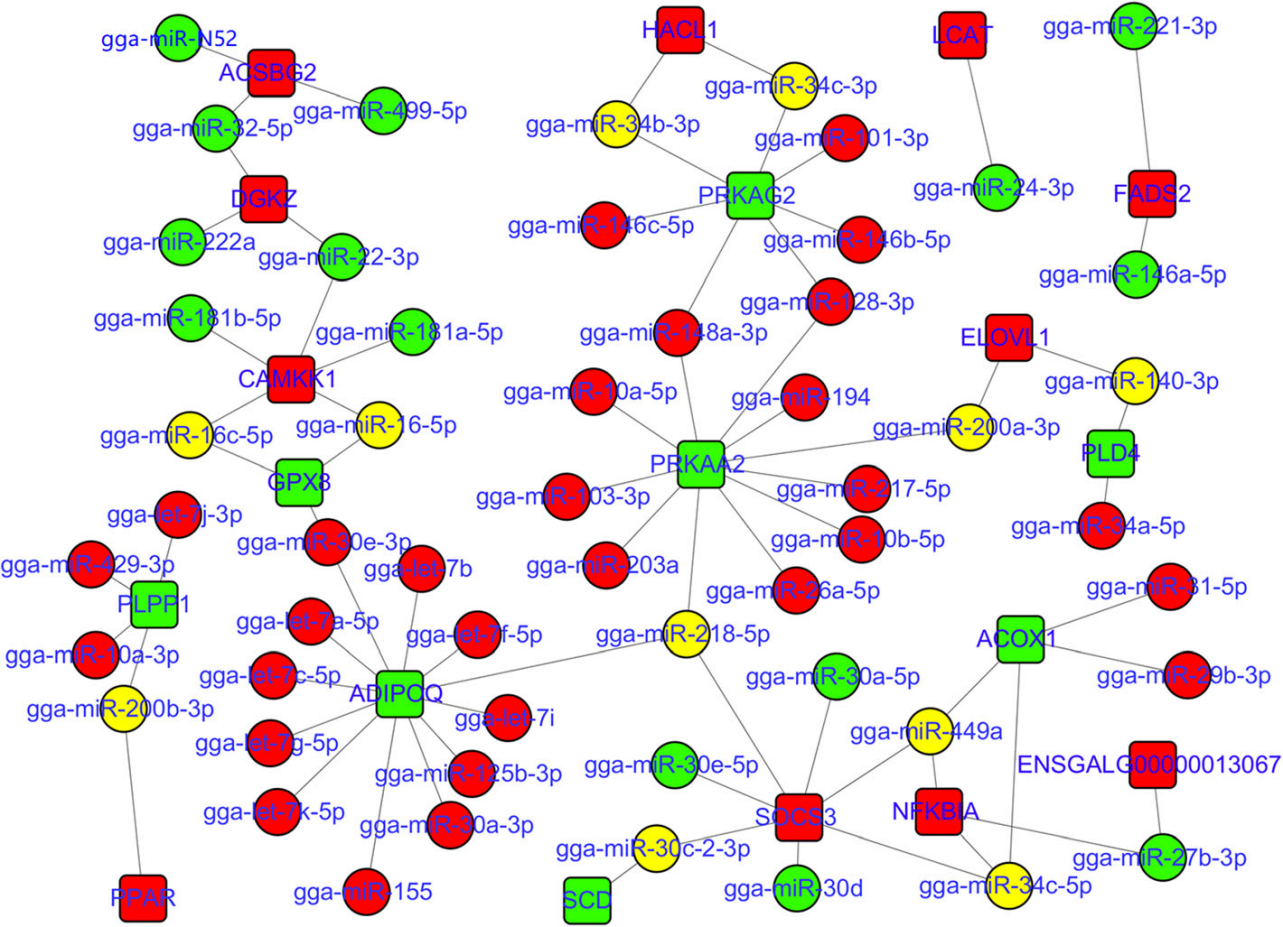
The miRNA–mRNA interaction networks containing eleven lipid metabolism or deposition related pathways. The dot indicates miRNA, and the box indicates a target gene that had a negative correlation with a given miRNA. Red indicates up-regulated, green indicates down-regulated, and yellow indicates a miRNA that was up-regulated in a given comparison combination and was down-regulated in other comparison combinations. The eleven pathways are fatty acid degradation, fatty acid metabolism, biosynthesis of unsaturated fatty acids, alpha-linolenic acid metabolism, fatty acid biosynthesis, arachidonic acid metabolism, fatty acid elongation, glycerophospholipid metabolism, glycerolipid metabolism, the adipocytokine signaling pathway and the PPAR signaling pathway. The miRNA–mRNA pairs in each pathway are shown in Additional file 9: Table S3

**Fig. 6:**
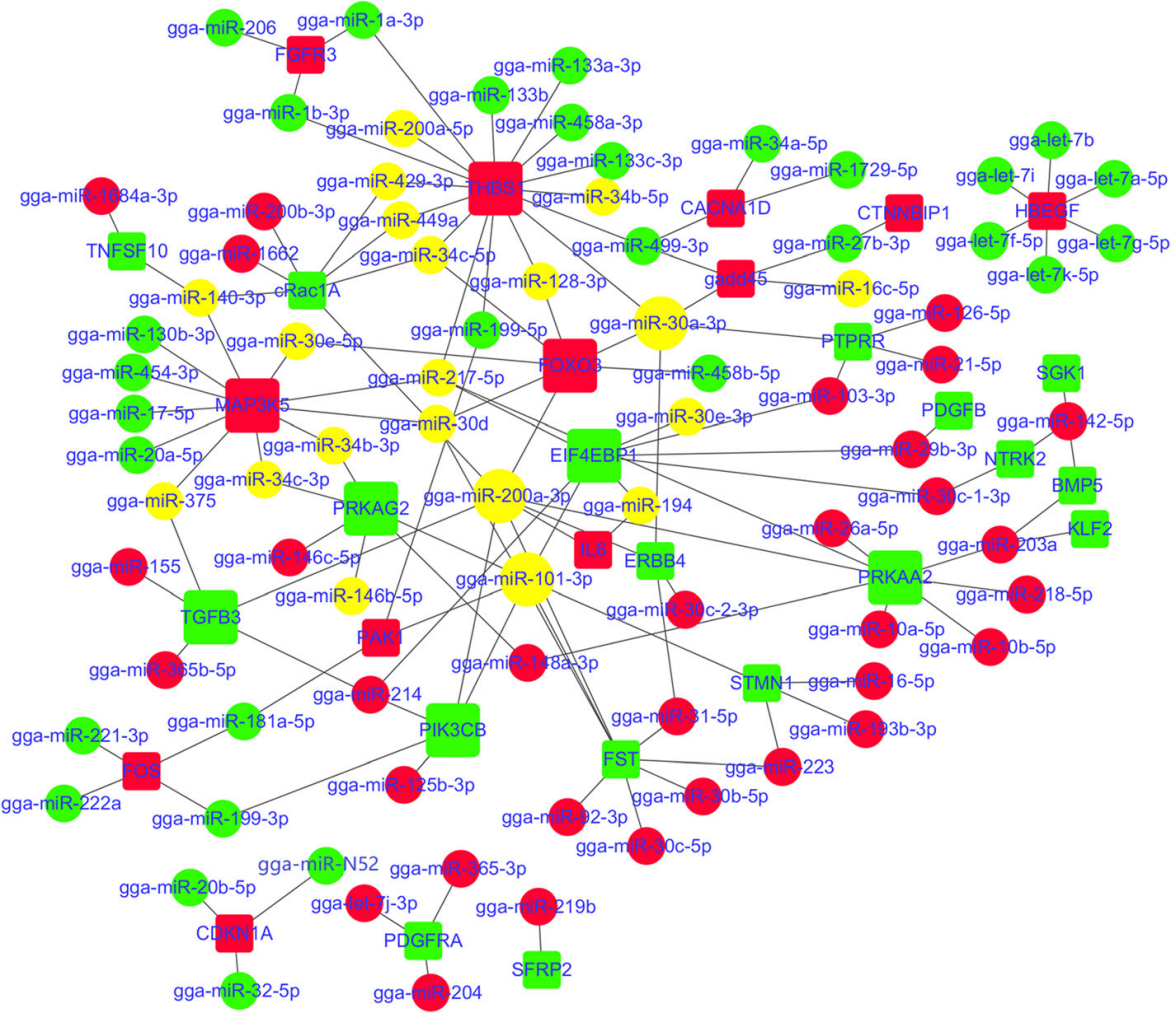
The miRNA–mRNA interaction networks containing five cell proliferation and differentiation related pathways. The dot indicates miRNA, and the box indicates a target gene that had a negative correlation with a given miRNA. Red indicates up-regulated, green indicates down-regulated, and yellow indicates a miRNA that was up-regulated in a given comparison combination and was down-regulated in other comparison combinations. The five pathways are FoxO signaling pathway, MAPK signaling pathway, TGF-beta signaling pathway, ErbB signaling pathway and Wnt signaling pathway. The miRNA–mRNA pairs in each pathway are shown in Additional file 9: Table S3

**Fig. 7:**
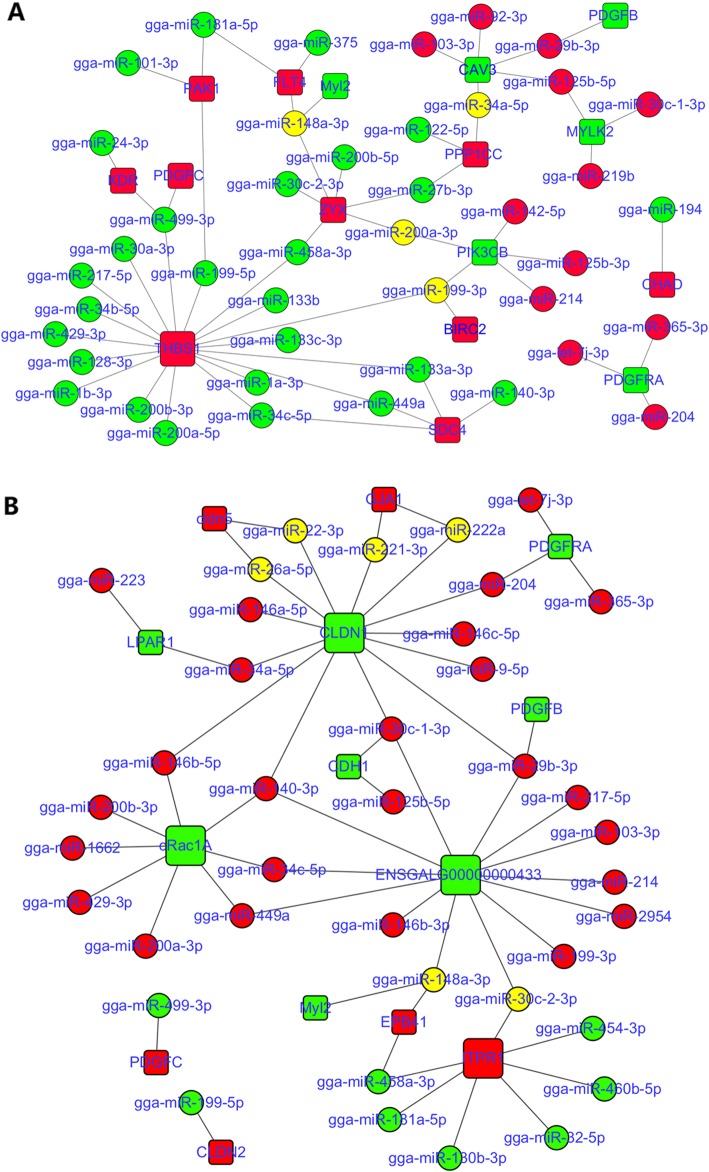
The miRNA–mRNA interaction networks containing two matrix connectivity (**a**) and (**b**) represent the miRNA–mRNA interaction networks with three cell junction (focal adhesion, ECM-receptor interaction, gap junction) and two matrix connectivity (tight junction, and adherens junction) related pathways, respectively. The dot indicates miRNA, and the box indicates a target gene that had a negative correlation with a given miRNA. Red indicates up-regulated, green indicates down-regulated, and yellow indicates a miRNA that was up-regulated in a given comparison combination and was down-regulated in other comparison combinations. The five pathways are focal adhesion, ECM-receptor interaction, gap junction, tight junction, and adherens junction. The miRNA–mRNA pairs in each pathway are shown in Additional file 9: Table S3

A total of 15,756 genes expressed in all four developmental stages were identified from twelve cDNA libraries of Gushi chicken abdominal fat. Under the criteria of the corrected *P*-value < 0.05, 794, 944, 966, 229, 806 and 733 differentially expressed genes were obtained from six combinations, respectively (Additional file 5: Figure S5). Based on these transcriptome data and miRNA expression profiles, 1543, 1859, 232, 1659, 1607 and 1331 differentially expressed miRNA-mRNA pairs were identified from the combinations of z14 vs. z06, z22 vs. z06, z30 vs. z06, z22 vs. z14, z30 vs. z14, and z30 vs. z22, respectively. The enriched pathways of these miRNA–mRNA pairs were significantly different in the different combinations (Additional file 6: Figure S6). We focused on twenty-one pathways that were closely related to abdominal fat development (Additional file 14). These pathways were involved in fat deposition, glycerolipid metabolism, fatty acid metabolism, cell junctions, cell proliferation and differentiation, and they were enriched in all six combinations. The miRNA–mRNA interaction networks were constructed using the negatively correlated miRNA–mRNA pairs selected from the above pathways (Figs. 5, 6, and 7). These networks contained a total of 98 miRNAs. In particular, members of some miRNA families such as let-7, mir-30, mir-10, mir-8, mir-221, mir-34, mir-199, mir-499, mir-458, mir-146, and mir-365 made up the vast majority, which acted on multiple pathways, respectively, and exhibited complex interactions.

In the miRNA–mRNA interaction networks associated with lipid metabolism or deposition (Fig. 5), some genes, such as *ACSBG2*, *ACOX1*, *ADIPOQ*, *SCD*, *PRKAG2*, *FADS2*, *DGKZ*, *PRKAA2*, *HACL1*, *PLPP1*, and *SOCS3*, as well as some miRNAs, such as gga-miR-449a, gga-miR-34c-5p, gga-miR-32-5p, gga-miR-200a-3p, and gga-miR-N52, constituted the key nodes of these networks. Among the miRNAs in these networks, members of the mir-8, mir-34, and mir-30 families act on nine, eight, and five pathways, respectively, and they appear to have a wide range of regulatory effects. Moreover, some miRNAs, such as gga-miR-140-3p, gga-miR-146a-5p, gga-miR-16-5p, gga-miR-200a-3p, gga-miR-221-3p, gga-miR-29b-3p, gga-miR-31-5p, gga-miR-32-5p, gga-miR-449a, gga-miR-499-5p, and gga-miR-N52, mainly act on the pathways related to fatty acid degradation, alpha-linolenic acid metabolism and biosynthesis of unsaturated fatty acids. It is noteworthy that the gene *ADIPOQ* in the PPAR signalling pathway and adipocytokine signalling pathway is targeted by seven members of the let-7 family.

Other members of the mir-8, mir-30 and mir-34 families in the miRNA–mRNA interaction networks were associated with cell proliferation and differentiation (Fig. 6), and they acted on four or more pathways, respectively. Moreover, miR-199, miR-217-5p, miR-449a, and miR-499-3p also target three or more pathways, respectively. In contrast, miRNAs such as gga-miR-10a-5p, gga-miR-146b-5p, and gga-miR-146c-5p only target the FoxO signalling pathway, while some miRNAs, including gga-miR-1729-5p, gga-miR-193b-3p, gga-miR-206, gga-miR-21-5p, gga-miR-222a, and gga-miR-499-5p, only act on the MAPK signalling pathway. Therefore, these miRNAs have specific roles.

Similarly, in the miRNA–mRNA interaction networks associated with matrix and cell junctions (Fig. 7), members of the mir-199, mir-30, mir-34, mir-8, and mir-146 families also have a wide range of regulatory roles, and they act on five, five, four, and three pathways, respectively. The mir-8 family mainly acts on the pathways related to matrix connectivity, while the mir-146 family mainly targets the pathways associated with cell junctions. In contrast, miRNAs such as gga-miR-133b, gga-miR-1a-3p, gga-miR-200a-5p, gga-miR-219b, gga-miR-24-3p, gga-miR-34b-5p, and gga-miR-92-3p only target focal adhesion, while some miRNAs, including gga-miR-146b-3p, gga-miR-223, and gga-miR-460b-5p, only act on the adherens junction.

## Discussion

Approximately 25–70% of body fat distribution changes are influenced by genetic background in animals. In chickens, abdominal fat weight (AbFW) and abdominal fat percentage (AbFP) are often used as phenotypic indices of fat traits. The heritability coefficients (*h*^*2*^) are 0.62 for AbFW and 0.24 for AbFP. However, these traits are genetically different in different chicken breeds and lines. In fact, the *h*^*2*^ of abdominal fat mass varies from 0.5 to 0.8 in chicken [33]. This finding suggests that it should be feasible to modulate abdominal fat deposition in chickens using genetic control strategies. In poultry production, excessive abdominal fat is often removed as a poultry by-product because it affects meat quality. Today, genetic selection for low-abdominal fat has become the main goal of meat-type chicken breeding. Therefore, investigations into the molecular mechanisms of abdominal fat deposition in chickens are important for increasing productivity. In the last few decades, there have been extensive studies on the cellular basis, as well as regulatory gene identification and the expression profile of abdominal adipose tissue in chickens [2, 33–38]. However, knowledge of post-transcriptional regulatory mechanisms underlying abdominal adipose tissue development in chickens, particularly the role and function of miRNAs, is still very limited. In this study, we constructed dynamic expression profiles of miRNAs in the abdominal adipose tissue of Gushi chickens at 6, 14, 22, and 30 weeks and identified 507 known miRNAs and 53 novel miRNAs. These results expand the number of miRNAs expressed in abdominal adipose tissue and provide a valuable resource for the selective breeding of abdominal fat traits in chickens.

According to our previous studies, the abdominal fat rates (i.e., abdominal fat weight/live weight× 100%) of Gushi chickens at 6, 14, 22 and 30 weeks were 0.63 ± 0.42, 1.95 ± 1.08, 3.25 ± 1.26 and 2.96 ± 0.35, respectively. In particular, the period from 14 weeks to 22 weeks is the rapid deposition stage of abdominal fat in Gushi chicken. Functional enrichment analysis of differentially expressed miRNAs was completed in this study. The results showed that the enriched biological processes were significantly different at different developmental stages of Gushi chicken abdominal fat. The biological processes comprising the regulation of apoptotic process, cell cycle process, and regulation of cellular component organization were mainly enriched between 6 weeks and 14 weeks, and the biological process of macromolecular complex disassembly was enriched between 14 weeks and 22 weeks, while the biological process of fatty acid metabolism was significantly enriched between 22 weeks and 30 weeks (Fig. 4). This finding suggests that 14 weeks and 22 weeks are important stages in abdominal fat deposition in Gushi chicken. Generally, the abdominal fat mass is determined by the number and volume of adipocytes in the depot [39]. It is speculated that in Gushi chicken, the abdominal fat pad and adipocyte hyperplasia dominate before 14 weeks, and adipocyte hypertrophy dominates between 14 weeks and 22 weeks, while growth of abdominal fat is primarily the filling of existing adipocytes with lipids after 22 weeks. The dynamic change in abdominal fat deposition in Gushi chicken is largely consistent with the cellular basis of abdominal adipose tissue development described in previous studies [2]. Therefore, the miRNA dynamic expression profiles obtained in this study reflect the mechanisms of molecular regulation of abdominal adipose tissue development at the post-transcriptional level in Gushi chicken.

Previous studies have confirmed that many miRNAs are expressed in a tissue-specific manner. In this study, 45 miRNA families with more than two members were detected, and most of the members in these miRNA families were expressed in high abundance in Gushi chicken abdominal adipose tissue. The most abundant miRNAs were the let-7 family, of which nine members were detected in Gushi chicken abdominal fat. In particular, six members, including let-7a-5p, let-7b, let-7f-5p, let-7 g-5p, let-7i, and let-7 k-5p, were expressed in high abundance in abdominal fat at four developmental stages. Let-7 has been reported to directly target the high-mobility group AT-hook 2 (*HMGA2*) and to inhibit adipocyte differentiation [15]. Of these abundant miRNAs, the miRNA with the highest abundance was gga-miR-148a-3p. MiR-148a has previously been shown to promote adipogenesis by targeting *WNT1* [40]. It is known that the miR-17–92 miRNA cluster promotes adipocyte differentiation by targeting RB2/p130 [9]. As a member of the miR-17–92 cluster, miR-17–3p was previously found to enhance 3 T3-L1 differentiation by targeting fatty acyl desaturase genes [41]. Interestingly, six members of the mir-17 family were found in Gushi chicken abdominal adipose tissue. Similarly, other abundant miRNAs, miR-30a-5p, miR-146b-5p, miR-30d, miR-21, miR-101-3p, and miR-27b-3p, have been shown to regulate adipogenesis in other species [10, 14, 42–44]. Moreover, some miRNAs that showed developmental stage-specificity were also found in Gushi chicken abdominal adipose tissue (Additional file 11). Although they were expressed in low abundance in Gushi chicken abdominal fat, some of them, such as miR-103, miR-31, and miR-135a, have previously been reported to participate in the regulation of adipocyte differentiation or adipogenesis [45–49]. These results suggest that developmental stage-specificity and high-abundance miRNAs may play an important role during abdominal adipose tissue development in chickens.

Many miRNAs are expressed in a spatiotemporal-specific manner in organisms. In this study, 51 significantly differentially expressed (SDE) miRNAs were screened from Gushi chicken abdominal adipose tissue at four developmental stages. These SDE miRNAs showed obvious temporal expression characteristics, and their expression patterns were divided into six types (Fig. 2). Specifically, three miRNAs, miR-215-5p, miR-122-5p and miR-499-5p, were differentially and abundantly expressed across the four stages during abdominal adipose tissue development. MiR-122 is a liver tissue-specific miRNA and plays a role in lipid metabolism [50]. Recent research has shown that miR-122 overexpression induces hepatic differentiation of adipose tissue-derived stem cells [51]. In addition, miR-122 is also the most significant signature between visceral fat and subcutaneous fat, and its abundance affects PPAR-γ signalling and adipocyte differentiation in vitro and in human adipose tissues [52]. Although the function of miR-499 in lipid metabolism has not been reported, a recent study has shown that miR-499 can regulate *PRDM16* to affect insulin-induced skeletal muscle satellite cell (SMSC) adipogenic differentiation [53]. Moreover, previous studies have suggested that miR-215-5p is a negative regulator during early adipogenesis in 3 T3-L1 cells [54]. For the above three miRNAs, further studies are required to clarify the relationship between their expression and fat-related phenotype and between the modulations of their expression and fat content, which will ultimately determine whether they can serve as molecular markers of chicken abdominal adipose tissue development. In addition to the above three miRNAs, other SDE miRNAs, miR-29b-3p, miR-1a-3p, miR-10a, miR-206, miR-429, miR-200a, miR-454, miR-200b, miR-31-5p, miR-204, miR-375, miR-155, miR-194, miR-130, and miR-365, have previously been shown to be related to adipogenesis [11, 13, 17, 55–59]. miR-375 has been reported to promote adipogenesis by extracellular signal regulated kinase (*ERK1/2*) signalling [13]; miR-204 has been found to improve adipogenesis by targeting runt-related transcription factor 2 (*RUNX2*) [11]. Conversely, miR-130 has been reported to directly target *PPAR-γ* and to inhibit the differentiation of human pre-adipocytes [17]. Similarly, miR-155 has been demonstrated to inhibit adipogenesis by targeting the cAMP response element binding protein (*CREB*) and *C/EBPβ* [59]. The above results indicate that these temporally and differentially expressed miRNAs are closely related to abdominal fat development in chickens.

In organisms, miRNAs construct a sophisticated control network and participate in the regulation of various biological processes through complex interactions with their target genes. To reveal the potential regulatory relationships of miRNAs underlying abdominal adipose tissue development in Gushi chicken, we carried out correlation analysis between miRNA and mRNA and performed KEGG pathway enrichment analysis of differentially expressed miRNA–mRNA pairs. In addition, based on the results of KEGG pathway analysis, we constructed miRNA–mRNA interaction networks involved in fatty acid metabolism, glycerolipid metabolism, cell junctions, and cell proliferation and differentiation during abdominal adipose tissue development in Gushi chicken (Figs. 5, 6 and 7). In these miRNA–mRNA interaction networks, some key genes as components of multiple pathways link multiple pathways to each other. For instance, *ACSBG2*, *ACOX1* and *FADS2* are found across five pathways, including fatty acid metabolism, fatty acid degradation, PPAR signalling pathway, biosynthesis of unsaturated fatty acids and alpha-linolenic acid metabolism, and *ADIPOQ* is found in the adipocytokine signalling pathway and PPAR signalling pathway. Similarly, *THBS1* also connects three pathways: the focal adhesion, transforming growth factor (TGF)-beta signalling pathway and ECM-receptor interaction. In these interaction networks, some miRNAs regulate the functions of multiple pathways by targeting multiple genes. Thus, a complex regulatory network formed by the interactions between miRNAs and their target gene and between pathways regulates abdominal adipose tissue development in Gushi chicken. After exhaustive literature mining of the miRNAs constituting the abovementioned interaction network, some miRNAs, including miR-148a, miR-103, miR-31, miR-204, miR-375, miR-155, miR-130, miR-142, miR-101, miR-200, miR-29b, miR-9, miR-32, miR-222, miR-206, miR-1a, miR-146b, miR-181, miR-30, miR-22, miR-27, and miR-194, as well as let-7 family members and the miR-17–92 cluster, have previously been reported to be related to lipid metabolism and adipogenesis [9, 11, 13–17, 40, 42–45, 47, 55–57, 59–64]. Therefore, these interactive networks reflect the complexity of molecular regulation of abdominal adipose tissue development at the post-transcriptional level in chickens.

Adipocyte hyperplasia and hypertrophy involve a series of cellular events. To better understand the post-transcriptional regulation mechanisms related to different cellular events during abdominal adipose tissue development in chickens, we constructed the miRNA–mRNA interaction networks of ten pathways involved in the matrix interaction, cell junction, and cell proliferation and differentiation (Figs. 6 and 7). We found that the *THBS1* gene connecting the focal adhesion, ECM-receptor interaction and TGF-beta signalling pathway was targeted by more than sixteen miRNAs, which mainly belonged to the mir-133, mir-199, mir-1, mir-200 and mir-34 families. In particular, miR-1 was reported to directly target the *THBS1* gene in previous studies [65]. Moreover, the key gene *TGFB3* connecting the TGF-beta signalling pathway, FoxO signalling pathway and MAPK signalling pathway was targeted by gga-miR-155, gga-miR-200a-3p, gga-miR-214, gga-miR-365b-5p and gga-miR-375, respectively. MiR-155 has been demonstrated to inhibit adipogenesis [59]. In these networks, some miRNAs targeting multiple target genes form complex interactions in multiple pathways, including the mir-146, mir-200, mir-30 and mir-34 family members, as well as miR-101, miR-103, miR-125b, miR-128, miR-181a, miR-140, miR-194, miR-199, miR-204, miR-148a, miR-214, miR-217, miR-221, miR-222a, miR-27b, miR-29b, miR-375, miR-429, miR-449a, miR-458a, and miR-499. Most of these miRNAs have been demonstrated to play an important role in adipogenesis or lipid metabolism. For instance, miR-27a/b [14, 66, 67] and miR-130 [17] have been demonstrated to target *PPAR-γ*. MiR-27a and mir-27b have been shown to regulate mouse and human adipogenesis [14, 66–69]. In addition, miR-146b has previously been demonstrated to affect adipogenesis by targeting *KLF7* [43] and *SIRT1* [70]. Interestingly, the interaction between miR-29b and *PDGFB* in focal adhesion and the MAPK signalling pathway [71], miR-101 and *PIK3CB* in the FoxO signalling pathway and ErbB signalling pathway [72], as well as miR-101 and *STMN1* in the MAPK signalling pathway [73], have been confirmed in previous studies.

The late development of abdominal adipose tissue is characterized by enhanced lipid metabolism and increased deposition of cytoplasmic triglycerides in chickens. In this study, we constructed the miRNA–mRNA interaction networks of eleven pathways involved in adipogenesis, fatty acid metabolism, and triglyceride metabolism (Fig. 5). It is known that the PPAR signalling pathway and adipocytokine signalling pathway are closely related to adipogenesis and lipid metabolism. We found that the key gene *ADIPOQ* connecting these two pathways was targeted by twelve miRNAs, including let-7a-5p, let-7b, let-7c-5p, let-7f-5p, let-7 g-5p, let-7i, let-7 k-5p, miR-125b-3p, miR-155, miR-218-5p, miR-30a-3p and miR-30e-3p. Interestingly, compared to wild-type adipocytes, miR-155 knockout adipocytes can upregulate the *ADIPOQ* gene in miR-155 KO mice [74], which demonstrates the interaction between miR-155 and *ADIPOQ*. In addition, the interaction between mir-181 family members and *CAMKK1*, miR-218 and *SOCS3*, as well as mir-30 family members and *SOCS3*, in the interaction network associated with the adipocytokine signalling pathway have also been confirmed in previous relevant studies [75–78]. *PPAR-γ* is a key transcription factor in adipocyte differentiation. We found that PPAR was targeted by miR-200b-3p in the interaction network associated with the PPAR signalling pathway. MiR-200b-3p has been reported to activate the PPAR-γ signalling pathway in diabetic cardiomyopathy [79], and its overexpression can decrease the expression levels of *PPAR-γ* in 3 T3-L1 cells [80]. However, the direct target relationship between miR-200b-3p and *PPAR-γ* still requires further verification. The glycerolipid metabolism and glycerophospholipid metabolism pathways have similar components, which were targeted by miR-222a, miR-22-3p, miR-32-5p, miR-34b-3p, miR-140-3p, let-7j-3p, miR-10a-3p, miR-200b-3p, miR-429-3p, and miR-24-3p, respectively. Three key genes, *ACSBG2*, *ACOX1* and *FADS2*, connect five pathways related to fatty acid metabolism, degradation and biosynthesis, as well as biosynthesis of unsaturated fatty acids and alpha-linolenic acid metabolism, were targeted by miR-499-5p, miR-32-5p, gga-miR-N52, miR-200a-5p, miR-31-5p, miR-34c-5p, miR-449a, miR-146a-5p and miR-221-3p, respectively. Most of these microRNAs have been shown to participate in lipid metabolism. In particular, the interaction between miR-31 and *ACOX1* in lipid metabolism has been confirmed in previous studies [81]. The above results reflect the complexity of the regulation of lipid metabolism during abdominal adipose tissue development at the post-transcriptional level in chickens.

## Conclusion

We have described the miRNA dynamic expression profiles and the miRNA–mRNA interaction networks involved in abdominal fat development in Gushi chicken. It was found that miRNAs regulate abdominal adipose tissue development, primarily by affecting lipid metabolism or deposition, adipocyte proliferation and differentiation, matrix interaction, and cell junctions, through a complex interaction network in chicken. These results provide novel insights and a valuable resource for a better understanding of the post-transcriptional regulatory mechanisms of abdominal fat development in chickens. The findings reported in this study will be further analysed and validated.

## Methods

### Animal feeding and sample collection

In this study, the experimental animals were Gushi chickens from the Animal Center of Henan Agricultural University. Female chickens were selected and raised in cages in the same environment with standard conditions. The diet comprised 18.5% crude protein and 12.35 MJ/kg energy before 14 weeks, and 15.6% crude protein and 12.75 MJ/kg energy after 14 weeks. Three healthy individuals were randomly selected at the ages of 6 weeks (z06), 14 weeks (z14), 22 weeks (z22) and 30 weeks (z30). These individuals were euthanized by intravenous injection of KCl (1–2 mg/kg) under deep anaesthesia. The abdominal fat pad and adipose tissue around the stomach were isolated and frozen immediately in liquid nitrogen. The frozen sample was stored at − 80 °C. Total RNA was isolated from abdominal adipose tissue using Trizol reagent (TaKaRa, China), and the mixed RNA samples from three individuals at each developmental stage were used for library construction.

### Small RNA library construction

Four small RNA libraries were constructed from abdominal adipose tissue of Gushi chicken at 6, 14, 22 and 30 weeks old using the Small RNA Sample Prep Kit (Illumina). First, 16–30-nt small RNAs were added to 3′ and 5′ adapters, and cDNA was reverse transcribed. Subsequently, PCR amplification was carried out, and the target DNA fragment was separated by PAGE gel electrophoresis, and the final cDNA library was obtained after enrichment. The library was diluted to 1 ng/μl, and then the library’s insert size was evaluated using an Agilent 2100 Bioanalyzer (Agilent Technologies, USA). Finally, the qualified cDNA library, i.e., The insert size met our expectation, and the effective concentration was more than 2 nM in the libraries and sequenced on the Illumina HiSeq 2500 sequencing platform.

### Post-sequencing analysis

The raw reads were processed to obtain clean reads by removing reads containing poly-N with 5′ adapter contaminants without 3′ adapter or the insert tag containing poly-A or T or G or C and low-quality reads. Then, the 18–35-nt clean reads were mapped to the chicken genome by Bowtie software [82]. The reads mapped onto the chicken genome were selected and blasted against the GenBank and Ensembl ncRNA databases, as well as the RNA families in Rfam, to annotate and remove the non-coding RNA sequences. The remaining sequences were then used to identify known miRNAs in the abdominal adipose tissue of Gushi chickens by comparing them with the mature chicken miRNAs in miRBase (Release 22.0). Finally, to identify potentially novel miRNAs in Gushi chicken abdominal fat, the remaining unannotated sRNA sequences were aligned against the chicken genome assembly (Release 4.0) from Ensemble to obtain genomic sequences containing sRNA. The hairpin structures and folding energy for the surrounding 300 bases flanking each small RNA sequence were predicted using miREvo [83] and miRDeep2 [84] software. The sequences with a typical stem-loop hairpin structure were considered potential novel miRNAs. These novel miRNAs were named as described by Ambros et al. (2003) [85].

### Identification and GO enrichment analysis of SDE miRNAs

The miRNA expression levels at different developmental stages of Gushi chicken abdominal fat were estimated using the transcripts per million clean reads (TPM). The following formula was used to calculate the normalized expression values: (read count × 1,000,000)/total miRNA read count in four libraries. If the normalized expression of a given miRNA is zero in all samples, this miRNA is removed in future differential expression analysis. The differentially expressed miRNAs were analysed by DEGseq [86]. The fold change for each miRNA between two discretionary developmental stages was calculated based on the comparison of six combinations, i.e., z06/z14, z06/z22, z06/z30, z14/z22, z14/z30, and z22/z30. Fisher’s test was used to determine the *P*-values, and the false discovery rate (FDR) method was introduced to determine the threshold *p* value in multiple tests [87]. MiRNAs with a q value < 0.01 and | log_2_ (fold change) | > 1 were considered SDE miRNAs.

To reveal the potential functions of SDE miRNAs during the development of Gushi chicken abdominal fat, miRanda [88] and TargetScan [89] were used to predict the potential target genes of the SDE miRNAs. For the miRanda software, all detected targets with scores and energies less than the threshold parameters of S > 140 and ΔG < -10 kcal/mol and strict 5′ seed pairing were selected as potential targets. For the TargetScan software, the 2–8-nt sequences that start from 5′ small RNA were chosen as seed sequences to predict the 3’UTR of mRNA, and the predicted targets were ranked based on the predicted repression or aggregate *P*_*CT*_ score of the longest 3′-UTR isoform. Finally, only the sites belonging to the top quartile of ranked predictions and present in several species, including *Gallus gallus,* were retained as true binding sites. To make the identification of target genes robust, miRNAs that were simultaneously predicted by two programs were selected for this study. Gene ontology (GO) enrichment analysis of the predicted targets was performed using the GOseq method [90]. To obtain a corrected P-value, the FDR method was used to adjust the P-value threshold of multiple tests [87]. When the corrected *P*-values were less than 0.05, GO terms were considered significantly enriched.

### Interaction analysis between miRNAs and mRNAs

To analyse the interactions between miRNAs and mRNAs, we also determined the dynamic transcriptome profiles of abdominal fat using the same tissue samples and the small RNA libraries. A total of 12 cDNA libraries were constructed from Gushi chicken abdominal fat tissues at 6, 14, 22 and 30 weeks using NEBNext® UltraTM Directional RNA Library Prep Kit for Illumina® (NEB, Ipswich, MA, USA) and were sequenced on an Illumina Hiseq 2500 platform. The raw sequences were deposited into the NCBI Sequence Read Archive under BioProject number PRJNA551368. Post-sequencing analyses, such as sequence analysis and differential expression analysis, were performed using the methods described by Li et al. (2019) [91].

Based on the abovementioned transcriptome profile data, the potential target genes of differentially expressed miRNAs were predicted using miRanda [88] and TargetScan [89] software according to the above described method, and these target genes were matched with the miRNAs. According to the expression levels of miRNAs and genes in Gushi chicken abdominal fat, the differentially expressed miRNA–mRNA pairs were identified by Pearson’s correlation analysis. Kyoto Encyclopedia of Genes and Genomes (KEGG) pathway enrichment analysis of all target genes in each combination was performed using KOBAS software [92] to identify the pathways associated with lipid metabolism and adipogenesis. Given the inverse correlation between miRNA and target expression patterns, the negatively correlated miRNA–mRNA pairs were selected from the above pathways, and their interaction networks were determined by Cytoscape 3.4 (http://www.cytoscape.org/).

### Quantitative real-time PCR (qRT-PCR) analysis

To verify the accuracy of the sequencing data, a primerScript™ RT reagent Kit (TaKaRa, Dalian, China) was used for reverse transcription, and BμLge-Loop™ miRNA qRT-PCR Primer specific for the differentially expressed miRNAs was designed by GenePharma (GenePharma, Shanghai, China). The qRT-PCR analysis was performed using the LightCycler® 96 instrument (Roche Applied Science). All reactions were repeated three times. With the U6 small nuclear RNA as an endogenous control, the relative expression levels were determined using the 2^-△△CT^ method. The *P*-value calculation was performed by the *t* test, and *P* ≤ 0.05 was considered to be a significant difference.

## Supplementary information

**Additional file 1:** **Figure S1.** Sequence length distribution in each library.

**Additional file 2:** **Figure S2.** Venn diagram (A) and shared case (B) of potential target genes of the SDE miRNAs among different combinations.

**Additional file 3:** **Figure S3.** Top 20 significantly enriched biological processes for the predicted target genes of SDE miRNAs in different combinations.

**Additional file 4:** **Figure S4.** Comparison of the biological processes of lipid metabolism regulated by the SDE miRNAs at different developmental stages of Gushi chicken abdominal adipose tissue.

**Additional file 5:** **Figure S5.** Number of differentially expressed mRNAs in different combinations.

**Additional file 6:** **Figure S6.** Top 20 enrichment pathways for the differentially expressed miRNA–mRNA pairs in different combinations.

**Additional file 7:** **Table S1.** Annotations of small RNAs derived from Gushi chicken abdominal fat.

**Additional file 8:** **Table S2.** Details of the novel miRNAs identified in this study.

**Additional file 9:** **Table S3.** Details of the significant differentially expressed miRNAs identified in this study.

**Additional file 10:** **Table S4.** The main biological processes associated with lipid metabolism or fat deposition during abdominal adipose tissue development in Gushi chicken.

**Additional file 11.** The read count and TPM value of miRNAs in each library.

**Additional file 12.** The results of miRNA family analysis.

**Additional file 13.** Different significantly enriched GO terms associated to the SDE miRNAs in the z14 vs. z06, z22 vs. z14 and z30 vs. z22.

**Additional file 14.** The miRNA-target gene pairs enriched in the pathway of interest.

## Data Availability

All data generated or analysed in this study are provided in additional files. The raw sequences from this study have been deposited to the NCBI Sequence Read Archive under accession number PRJNA528858 (BioProject ID of miRNA) and PRJNA551368 (BioProject ID of mRNA).

## Abbreviations

ACOX1: Acyl-CoA oxidase 1
ACSBG2: Acyl-CoA synthetase bubblegum family member 2
ADIPOQ: Adiponectin, C1Q and collagen domain containing
C/EBPβ: CCAAT/enhancer binding protein
CAMKK1: Calcium/calmodulin dependent protein kinase kinase 1
CREB: The cAMP response element binding protein
CTNNBIP1: Catenin, beta interacting protein 1
DGKZ: Diacylglycerol kinase zeta
ERK1/2: Extracellular signal regulated kinase
FADS2: Fatty acid desaturase 2
FNDC3B: Fibronectin type III domain containing 3B
HACL1: 2-hydroxyacyl-CoA lyase 1
HDAC6: Histone deacetylase 6
HMGA2: High-mobility group AT-hook 2
KLF7: Kruppel-like factor 7
PDGFB: Platelet derived growth factor subunit B
PHB: Prohibitin
PIK3CB: Phosphatidylinositol-4,5-bisphosphate 3-kinase catalytic subunit beta
PLPP1: Phospholipid phosphatase 1
PPAR-γ: Peroxisome proliferator activated receptor gamma
PRDM16: PR domain containing 16
PRKAA2: Protein kinase AMP-activated catalytic subunit alpha 2
PRKAG2: Protein kinase AMP-activated non-catalytic subunit gamma 2
RUNX2: The runt-related transcription factor 2
SCD: Stearoyl-CoA desaturase
SIRT1: Sirtuin 1
SOCS3: Suppressor of cytokine signaling 3
STMN1: Stathmin 1
TGFB3: Transforming growth factor beta 3
THBS1: Thrombospondin 1
WNT1: Wnt family member 1
