# Supplementary figures and images for: MicroRNAs and their regulatory networks in Chinese Gushi chicken abdominal adipose tissue during postnatal late development
Source: BMC Genomics. 2019 Oct 25;20:778. doi: 10.1186/s12864-019-6094-2 (PMC6815035; doi:10.1186/s12864-019-6094-2)

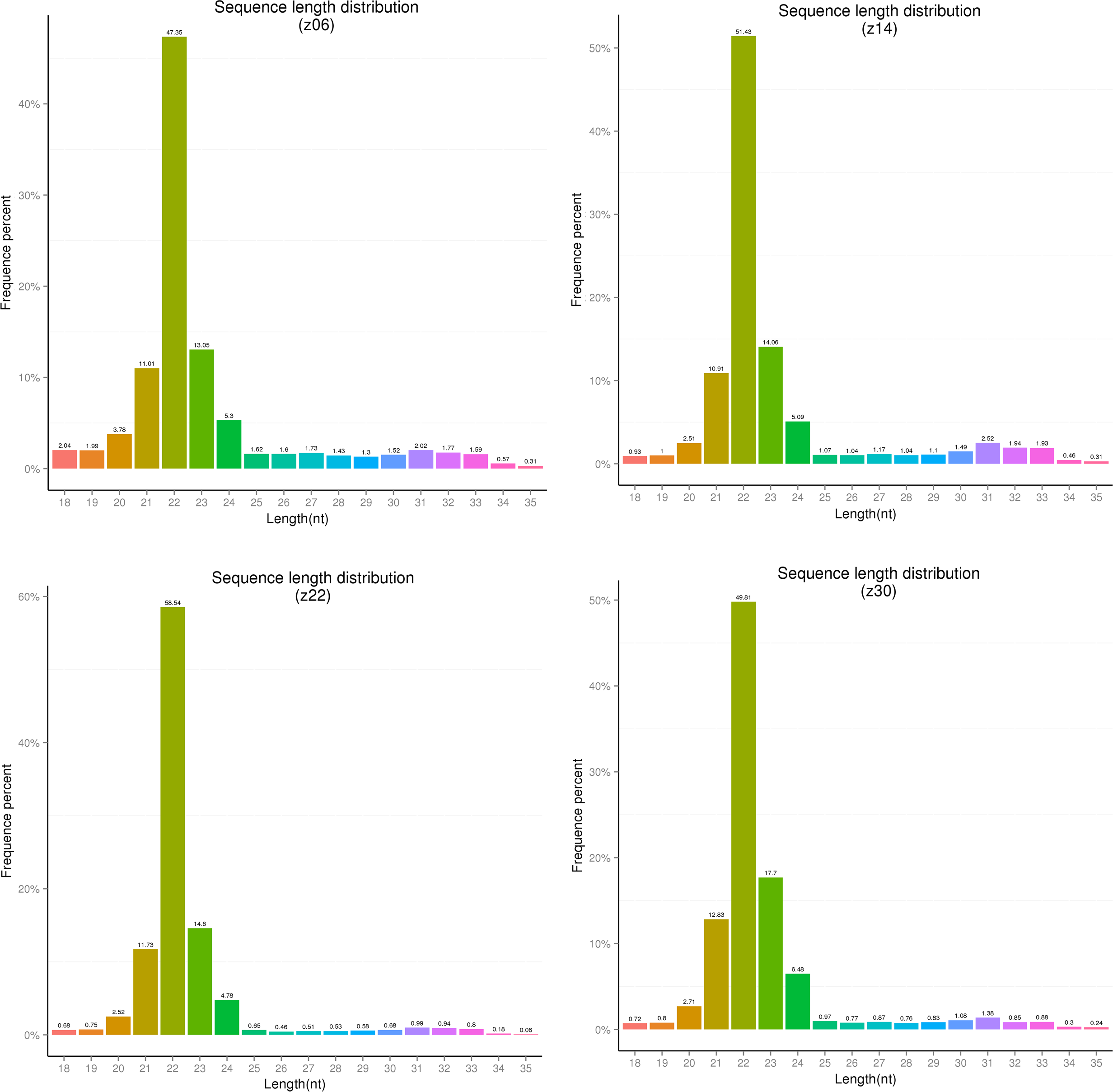

Supplement: Supplementary file 1 — Additional file 1: Figure S1. Sequence length distribution in each library. [file 12864_2019_6094_MOESM1_ESM.tif]

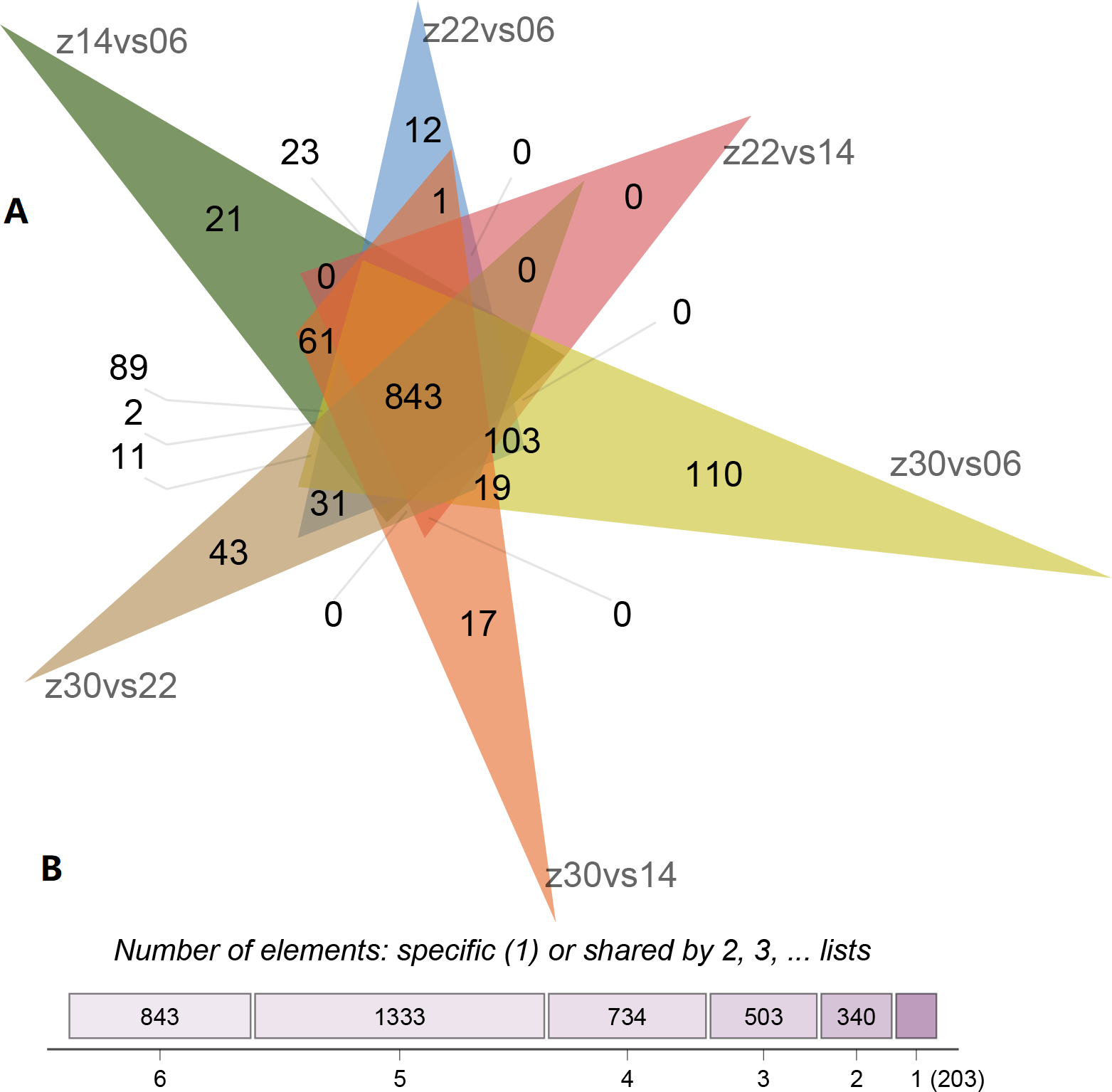

Supplement: Supplementary file 2 — Additional file 2: Figure S2. Venn diagram (A) and shared case (B) of potential target genes of the SDE miRNAs among different combinations. [file 12864_2019_6094_MOESM2_ESM.tif]

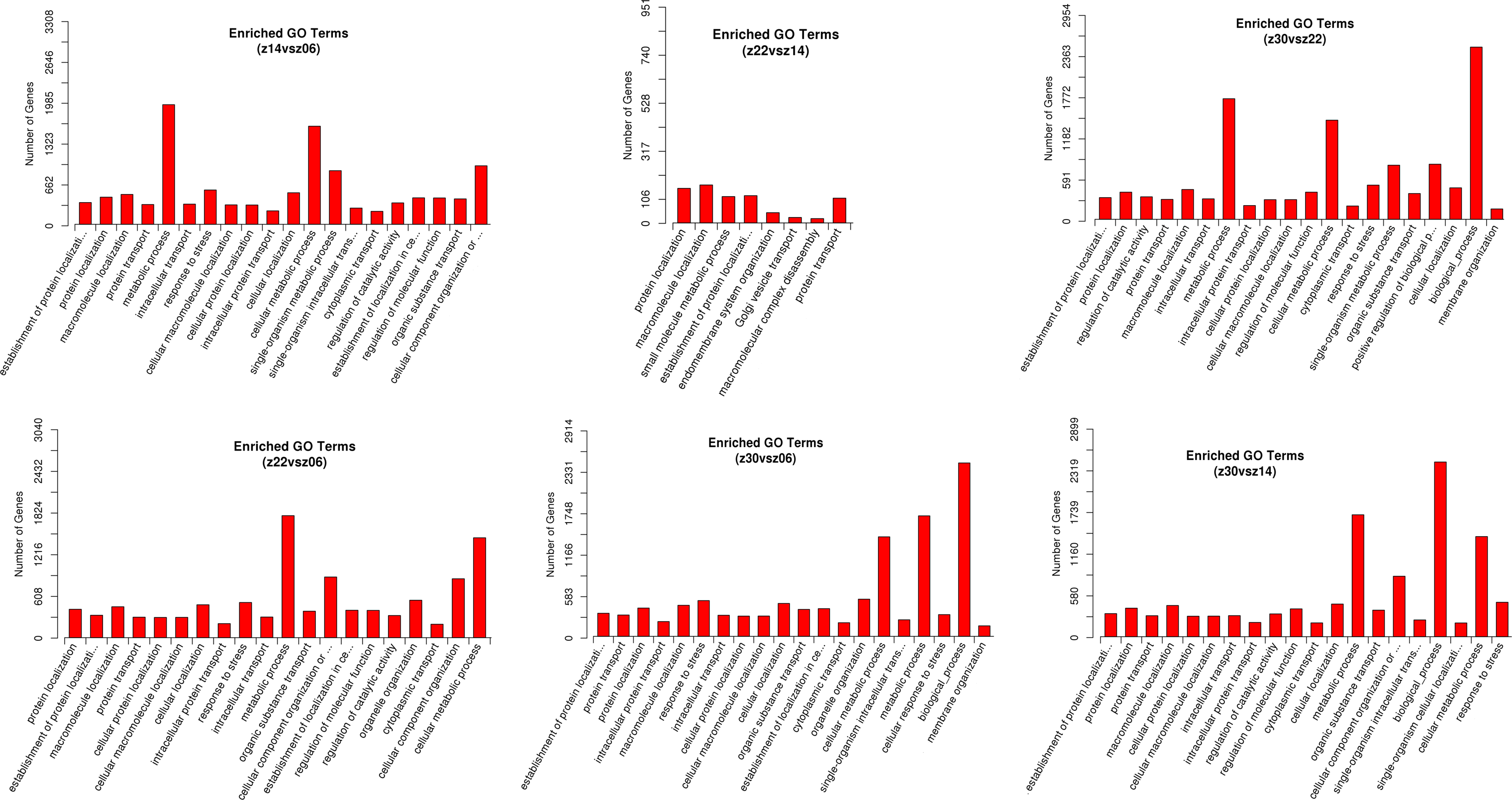

Supplement: Supplementary file 3 — Additional file 3: Figure S3. Top 20 significantly enriched biological processes for the predicted target genes of SDE miRNAs in different combinations. [file 12864_2019_6094_MOESM3_ESM.tif]

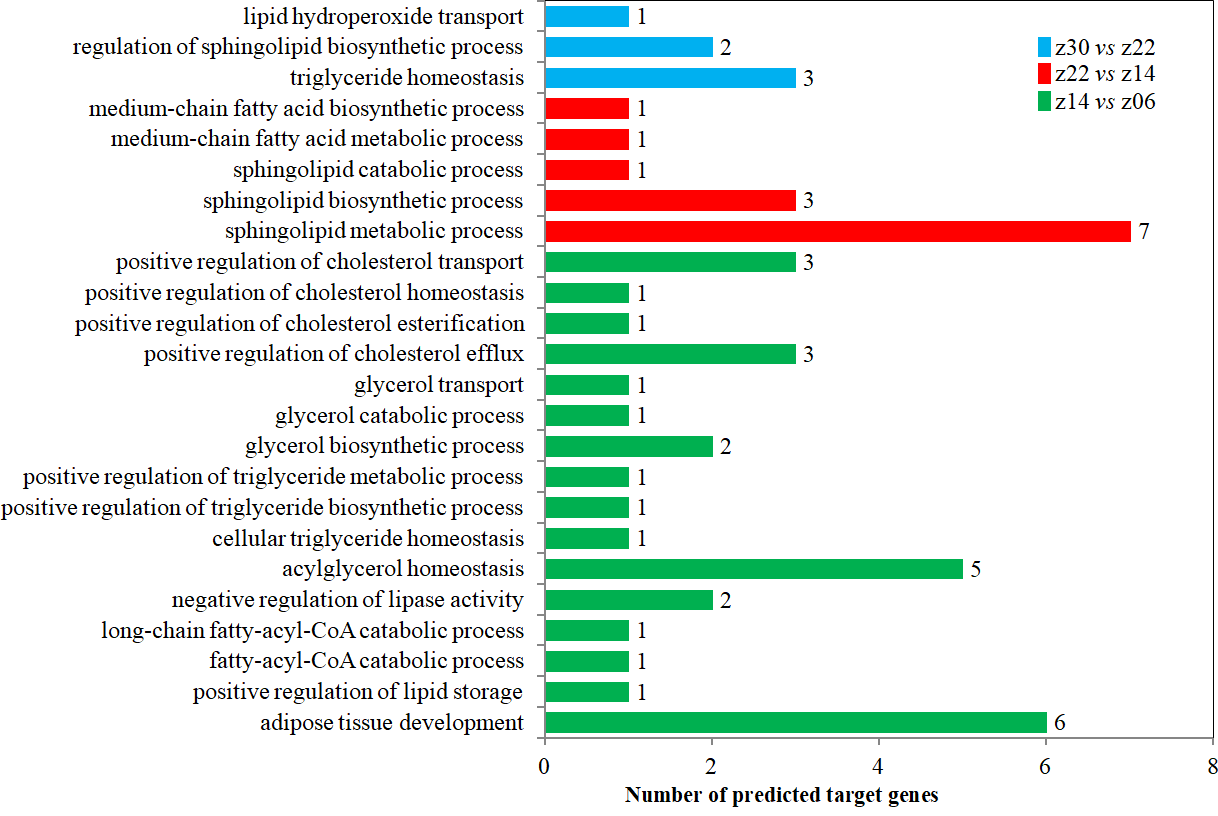

Supplement: Supplementary file 4 — Additional file 4: Figure S4. Comparison of the biological processes of lipid metabolism regulated by the SDE miRNAs at different developmental stages of Gushi chicken abdominal adipose tissue. [file 12864_2019_6094_MOESM4_ESM.tif]

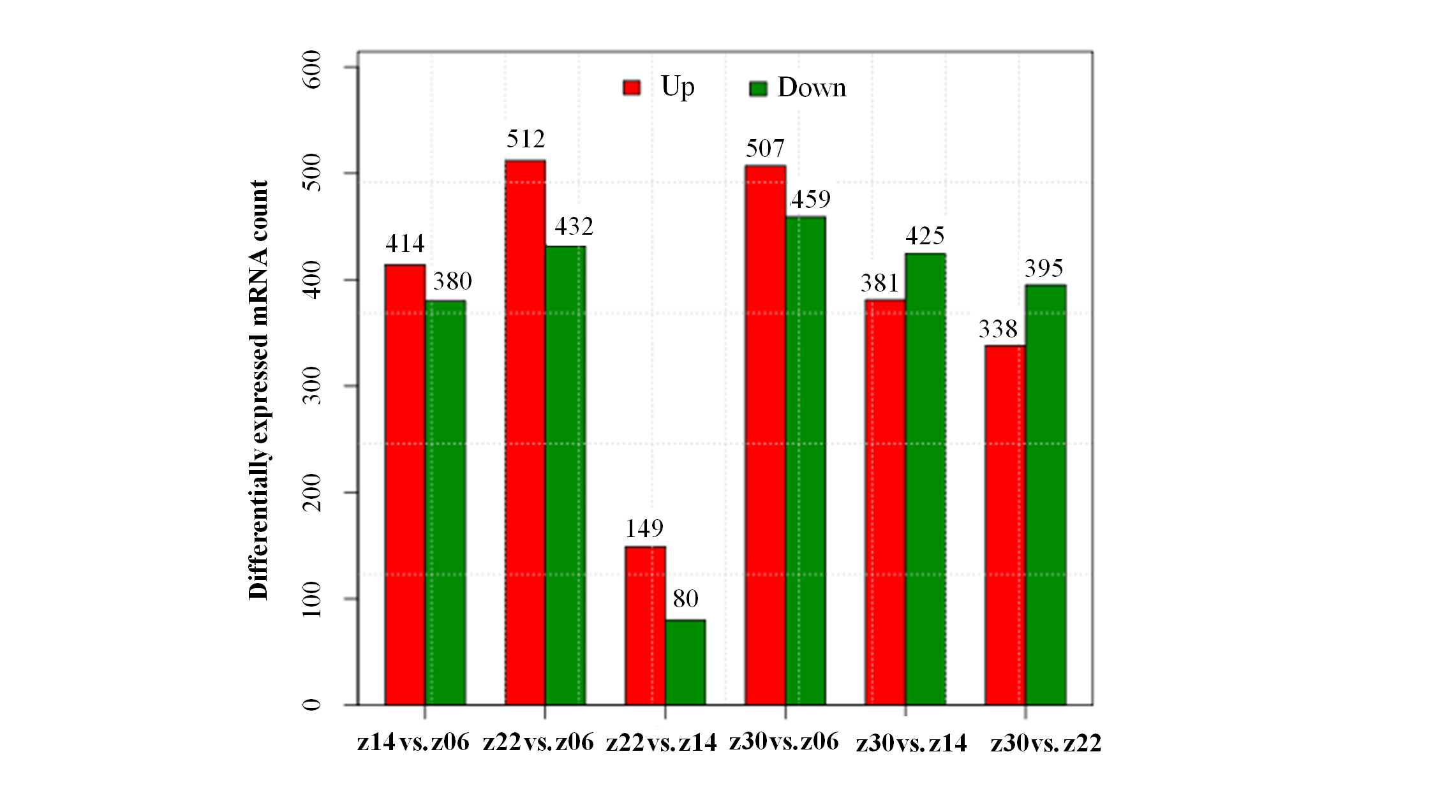

Supplement: Supplementary file 5 — Additional file 5: Figure S5. Number of differentially expressed mRNAs in different combinations. [file 12864_2019_6094_MOESM5_ESM.tif]

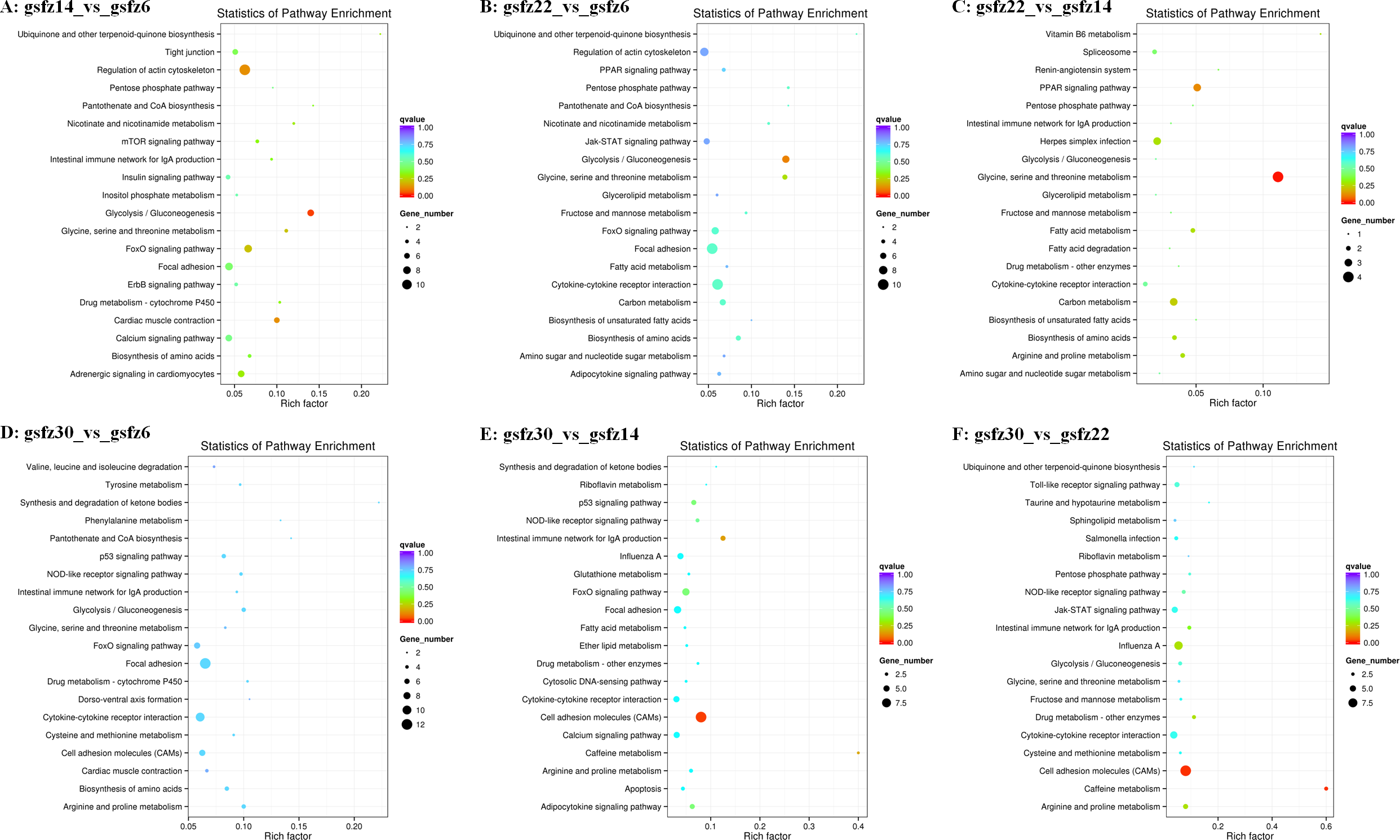

Supplement: Supplementary file 6 — Additional file 6: Figure S6. Top 20 enrichment pathways for the differentially expressed miRNA–mRNA pairs in different combinations. [file 12864_2019_6094_MOESM6_ESM.tif]
