# Supplementary material for: MicroRNAs and their regulatory networks in Chinese Gushi chicken abdominal adipose tissue during postnatal late development
Source: BMC Genomics. 2019 Oct 25;20:778. doi: 10.1186/s12864-019-6094-2 (PMC6815035; doi:10.1186/s12864-019-6094-2)
Supplement: Supplementary file 7 — Additional file 7: Table S1. Annotations of small RNAs derived from Gushi chicken abdominal fat. [file 12864_2019_6094_MOESM7_ESM.docx]

**Table S1** Annotations of small RNAs derived from Gushi chicken abdominal fat

| Types | z06 | | | | z14 | | | | z22 | | | | z30 | | | |
| --- | --- | --- | --- | --- | --- | --- | --- | --- | --- | --- | --- | --- | --- | --- | --- | --- |
|  | Count | Percent | Unique | Percent | Count | Percent | Unique | Percent | Count | Percent | Unique | Percent | Count | Percent | Unique | Percent |
| total | 9,083,101 | 100.00% | 929,903 | 100.00% | 21,630,786 | 100.00% | 1,075,445 | 100.00% | 11,414,249 | 100.00% | 323,834 | 100.00% | 11,765,740 | 100.00% | 449,550 | 100.00% |
| rRNA | 38,271 | 0.42% | 4,900 | 0.53% | 31,238 | 0.14% | 5,505 | 0.51% | 12,335 | 0.11% | 2,714 | 0.84% | 20,725 | 0.18% | 4,091 | 0.91% |
| tRNA | 5,685 | 0.06% | 1,263 | 0.14% | 13,284 | 0.06% | 2,018 | 0.19% | 2,180 | 0.02% | 670 | 0.21% | 8,458 | 0.07% | 1,711 | 0.38% |
| snRNA | 3,466 | 0.04% | 836 | 0.09% | 19,774 | 0.09% | 1,617 | 0.15% | 1,906 | 0.02% | 449 | 0.14% | 7,904 | 0.07% | 722 | 0.16% |
| snoRNA | 39,925 | 0.44% | 4,879 | 0.52% | 81,136 | 0.38% | 5,692 | 0.53% | 33,515 | 0.29% | 2,764 | 0.85% | 43,980 | 0.37% | 2,824 | 0.63% |
| repeat | 17,,735 | 0.20% | 16,715 | 1.80% | 24,864 | 0.11% | 20,745 | 1.93% | 6,428 | 0.06% | 5,850 | 1.81% | 9,232 | 0.08% | 8,060 | 1.79% |
| exon:+ | 686,821 | 7.56% | 203,656 | 21.90% | 1,486,505 | 6.87% | 301,639 | 28.05% | 734,326 | 6.43% | 67,255 | 20.77% | 901,761 | 7.66% | 128,754 | 28.64% |
| exon:- | 5,280 | 0.06% | 2,732 | 0.29% | 19,467 | 0.09% | 3,491 | 0.32% | 3,803 | 0.03% | 1,055 | 0.33% | 8,669 | 0.07% | 1,797 | 0.40% |
| intron:+ | 397,399 | 4.38% | 128,800 | 13.85% | 965,670 | 4.46% | 106,918 | 9.94% | 236,813 | 2.07% | 35,096 | 10.84% | 334,117 | 2.84% | 29,011 | 6.45% |
| intron:- | 27,777 | 0.31% | 7,214 | 0.78% | 22,241 | 0.10% | 7,744 | 0.72% | 8,274 | 0.07% | 2,913 | 0.90% | 11,949 | 0.10% | 4,628 | 1.03% |
| other | 4,136,374 | 45.54% | 556,220 | 59.81% | 9,450,433 | 43.69% | 616,601 | 57.33% | 5,114,070 | 44.80% | 202,387 | 62.50% | 4,853,965 | 41.26% | 265,010 | 58.95% |

Note: z06, z14, z22, and z30 represent small the RNA libraries obtained using samples from chickens aged 6, 14, 22, and 30 weeks, respectively.
