# Supplementary material for: MicroRNAs and their regulatory networks in Chinese Gushi chicken abdominal adipose tissue during postnatal late development
Source: BMC Genomics. 2019 Oct 25;20:778. doi: 10.1186/s12864-019-6094-2 (PMC6815035; doi:10.1186/s12864-019-6094-2)
Supplement: Supplementary file 8 — Additional file 8: Table S2. Details of the novel miRNAs identified in this study. [file 12864_2019_6094_MOESM8_ESM.docx]

**Table S2** Details of the novel miRNAs identified in this study

| miRNA ID | Mature miRNA sequence | sRNA read count | | | | Precursor sequence | Genome localization |
| --- | --- | --- | --- | --- | --- | --- | --- |
|  |  | **z06** | **z14** | **z22** | **z30** |  |  |
| gga-miR-N1 | uaagugcuggccguuguccucu | 23 | 117 | 74 | 74 | uaagugcuggccguuguccucuuaaguaccuguacuuagaggacaauggccaauacuugcu | 1:56587091..56587201:- |
| gga-miR-N2 | uuugggugccagccccguugcg | 11 | 7 | 5 | 9 | ggacggggcugggauccaggaccugcugcgggcauuggaaguguuuugggugccagccccguugcg | 5:388292..388403:-  10:14189122..14189232:- |
| gga-miR-N3 | uuucucaucgguggucugcucaga | 6 | 8 | 4 | 7 | uuugcagaccaccgaugagaacggaaaagauucccuuuucucaucgguggucugcucaga |  |
| gga-miR-N4 | ucaugcccucucugugccaggu | 0 | 1 | 0 | 0 | ugggcacaaagagggcagugggagcagggagagccucaugcccucucugugccaggu | 10:19601267..19601376:-  10:828017..828130:+ |
| gga-miR-N5 | ucagcaccccuccguccccgcagu | 0 | 5 | 1 | 5 | gcgggggcgcggggugcugagccguccuuuggggucucuccccgcgugggccuccccccgcucagcaccccuccguccccgcagu |  |
| gga-miR-N6 | auggcugugagugcggcugagg | 0 | 2 | 0 | 0 | auggcugugagugcggcugaggugucagagcauccuccucagccgucuugcagccuccu | 3:96067417..96067529:-  4:4111216..4111326:+ |
| gga-miR-N7 | cgccugggaggggagccgugu | 1 | 3 | 0 | 1 | ugggcccuccccugucaggcuggugguuuugaaucccccccgccugggaggggagccgugu |  |
| gga-miR-N8 | gcugggcuggugcaguugugc | 0 | 0 | 1 | 2 | auggcugcuccagucagguggugggauuucagugccgcugggcuggugcaguugugc | 21:3287226..3287336:-  19:2941332..2941444:- |
| gga-miR-N9 | aguguuaggugcagccuugcugc | 1 | 1 | 0 | 2 | aguaaggcuccuucugaccuccuugcacucucugggaccuggaguguuaggugcagccuugcugc |  |
| gga-miR-N10 | uuugaucuaaaggccacugacg | 1 | 2 | 1 | 0 | uuugaucuaaaggccacugacgucaaugagaguacuuccauugucuucagugggcuuuggaucaag | 8:25664316..25664427:+  LGE64:608008..608120:- |
| gga-miR-N11 | gauuguauucuuggcugagcuaac | 0 | 0 | 0 | 1 | gauuguauucuuggcugagcuaacaguauugccagcaggucaagaagaaauc |  |
| gga-miR-N12 | agcugccuggaugcucugcuggg | 2 | 0 | 0 | 0 | cugggagcagucagaggagcuaauuucuccagcugccuggaugcucugcuggg | 7:22120313..22120425:+ |
| gga-miR-N13 | auggcgcgggcgggcgggca | 2 | 2 | 2 | 6 | auggcgcgggcgggcgggcagaaggccgcugcucggaggagagcgcugcgccacuccccucggcucccgccggac | 1:33018217..33018326:-  20:9963060..9963171:- |
| gga-miR-N14 | cacuguauggugcuguacgcaug | 4 | 4 | 4 | 3 | cacuguauggugcuguacgcaugggcaggagaagggcauggggugcagcaccauacagugug |  |
| gga-miR-N15 | caucuuggaguauaagguaccug | 1 | 9 | 13 | 11 | caucuuggaguauaagguaccugauagagggugcagcagguaccugcaccccaagaugacu | 2:144736899..144737010:-  17:8397026..8397133:+ |
| gga-miR-N16 | gccuuggccggggcggggc | 0 | 2 | 1 | 0 | cccgccgccgcagggcaggaaagggcagagccgccaccgggccuuggccggggcggggc |  |
| gga-miR-N17 | ccaaagguggcucgcggaacuga | 0 | 2 | 0 | 2 | uuccgcgaaccgccuucggucgcggugucugcggagaccaaagguggcucgcggaacuga | 4:38970030..38970141:-  9:22081996..22082103:+ |
| gga-miR-N18 | ugugcucggcugaggagca | 2 | 0 | 0 | 1 | cucugauggcugcagcacgagcugugcucggcugaggagca |  |
| gga-miR-N19 | gcggcgccgggcggggcg | 0 | 1 | 0 | 0 | gcggcgccgggcggggcggagggagaggggccggcgcgagcgg | 7:9738809..9738916:- |
| gga-miR-N20 | aaugcuauggauucuguagga | 1 | 1 | 2 | 2 | aaugcuauggauucuguaggaauccacgcuguggaacacuccuacagaaucuauagcauuua | 4:77621259..77621369:+ |
| gga-miR-N21 | ucuggcugcucagugcucugcu | 2 | 0 | 0 | 2 | caggagcacugggugacgaggagccucgcacagcucuggcugcucagugcucugcu | JH375654.1:55992..56103:- |
| gga-miR-N22 | ugucccaccucugcccuccagc | 0 | 1 | 0 | 0 | uggaggugaccguggggagggcagguguaggaacugcaggcaaacgcagcccugucccaccucugcccuccagc | 11:979068..979178:-  2:138433729..138433839:+ |
| gga-miR-N23 | cugcugggaauacugaguaagg | 0 | 3 | 0 | 1 | uugcucaguauugcagacagcuguuacugucucuagcugcugggaauacugaguaagg |  |
| gga-miR-N24 | ggccauguugucugugggcucu | 1 | 5 | 1 | 2 | uacccacaaacaacauggcggcugugguuacaggcggccauguugucugugggcucu | 3:86130935..86131046:+  3:29623264..29623374:+ |
| gga-miR-N25 | ugccucuuugucuaucugcaga | 6 | 13 | 8 | 8 | ugcagguaggcagggggagcaggcugugugcugguugccuaccgucugccucuuugucuaucugcaga |  |
| gga-miR-N26 | cggacgaggcuucccgcgcugu | 1 | 2 | 2 | 0 | cgcgcgggaagccucggccgacgagcaaccggcggacgaggcuucccgcgcugu | 3:80936852..80936962:+  2:97400985..97401093:- |
| gga-miR-N27 | ccgggcgggagggagcggg | 0 | 4 | 1 | 1 | ccuccuucuccuccuccgccgggcugcggucccgccggcgcggacggcuccuccccggcggccgggcgggagggagcggg |  |
| gga-miR-N28 | acaacucugggcacucuggguccc | 22 | 20 | 23 | 4 | gcucaggaugcccagggccccauccaaccuggacaugagaaccuccagggauggggcacccacaacucugggcacucuggguccc | 10:12319538..12319650:+ |
| gga-miR-N29 | ucagccagggauugugggg | 0 | 1 | 0 | 0 | ucagccagggauuguggggagcacagagcgugcaguccuccaagcugcagacu | 22:2113247..2113354:+ |
| gga-miR-N30 | cugaugugugagcgcugugcacu | 1 | 4 | 0 | 4 | cugaugugugagcgcugugcacuggauccuauggaagugcccgugccucacgcucaagg | 1:120045022..120045133:- |
| gga-miR-N31 | agcuguacggcuggacauu | 2 | 0 | 0 | 0 | cuguucagcugugcaaugcgcuggcuagcuguacggcuggacauu | 9:15209040..15209148:- |
| gga-miR-N32 | ugcuccgucagacccaucagcagg | 1 | 2 | 1 | 2 | uauuccagcuggggucucucagcacagcuggcugcucgccguucggugcugcuccgucagacccaucagcagg | 8:27164758..27164870:- |
| gga-miR-N33 | ucuguagcauauaccagugaac | 1 | 3 | 2 | 2 | ucacugguauaugcuacagauuucagaaaagccauccaaaaucaaacaaucuguagcauauaccagugaac | 1:5580318..5580429:+ |
| gga-miR-N34 | gcggcucggcgcggcgcag | 0 | 4 | 0 | 0 | ccuccgcgcggagcagcggccacugccgagcgggcggcucgcugagugacagcgcggcucggcgcggcgcag | 20:12407407..12407514:+ |
| gga-miR-N35 | aucacaccgcugccacca | 0 | 3 | 5 | 1 | aucacaccgcugccaccagccacgggcaaaaccuacugaggaaaaugcagcggugcugguag | 4:24736141..24736248:- |
| gga-miR-N36 | cagcacagcacgggccuugagacg | 0 | 0 | 0 | 3 | uuuccaaggcuggaggaaggcaggaguccagccuccagcacagcacgggccuugagacg | 3:26736223..26736336:- |
| gga-miR-N37 | uugguggcugcaugcucucagc | 7 | 21 | 14 | 20 | ugagggcagagcugcagccucacauuucuucuuuuguuggacuugguggcugcaugcucucagc | 19:7369724..7369832:+ |
| gga-miR-N38 | ucagaaggcugcguguuc | 2 | 19 | 0 | 4 | acagugguuuacugacagucuauacuauauauauauuuguauguauacuggccguauaugcuguaucagaaggcugcguguuc | 4:75565478..75565585:+  1:179712225..179712332:+ |
| gga-miR-N39 | gugucuguagcucagcuga | 0 | 2 | 0 | 0 | agcauuccugcaagaaaccauugccucucuccaucucaucaagcuugaguggaggaagggaggugucuguagcucagcuga |  |
| gga-miR-N40 | ccaugguacugucugugc | 4 | 0 | 1 | 0 | acaugacauguucuuaguggggucgggagcuuguuagacagaggcaaggcccaugguacugucugugc | 2:61554920..61555027:- |
| gga-miR-N41 | acaacaggucugugaugcu | 3 | 4 | 1 | 0 | uauugccacaccuguugcaucaagguggcaacuucuaugcacacaacaggucugugaugcu | 20:12575125..12575232:+ |
| gga-miR-N42 | aguagagacuggauuuccugca | 18 | 45 | 9 | 35 | caggauauccucucuuggcauauuuucauuugccuaguuaguagagacuggauuuccugca | 18:6253585..6253696:- |
| gga-miR-N43 | ugugugcgugcguguaugucuga | 0 | 1 | 0 | 0 | ugugugcgugcguguaugucugaacacacgcagaccagcucacacucacacaca | 2:7411423..7411534:+  1:131038459..131038571:- |
| gga-miR-N44 | ucggcccugggagugucguucuu | 2 | 4 | 1 | 1 | caacggcaccuccgggcgccgcgggcugaaggcggcccucggcccugggagugucguucuu |  |
| gga-miR-N45 | ugcugaggagccacuggac | 1 | 0 | 0 | 0 | ugcugaggagccacuggacuguuuucugaauuuguggauuauucuuggcggu | Z:32651392..32651499:+ |
| gga-miR-N46 | aaccaagugggaaaguucucu | 2 | 0 | 0 | 0 | aggauuuuuccucuuugccuaacagcaucuccucugacugagcuaggcagacuucuuaggaaagaaaggaggugagaaaaccaaaaccaagugggaaaguucucu | Z:13418474..13418583:+  2:5210710..5210817:- |
| gga-miR-N47 | gcugcuuggcugaggagc | 2 | 0 | 0 | 0 | gcugcuuggcugaggagcaaaauaacuucauguauggcuucaugaauggcuccuuagcaaaccuggcuc |  |
| gga-miR-N48 | aaugauuugggcacugucug | 0 | 1 | 0 | 0 | aaugauuugggcacugucugaacaauggaauagaucaguucuaaaucagcuc | 3:36085389..36085497:- |
| gga-miR-N49 | ccaucuccaccagcagcacugu | 0 | 0 | 0 | 2 | agugccgacguguggggaugcgccguggggcagagcccaucuccaccagcagcacugu | 17:991798..991909:+ |
| gga-miR-N50 | auuccaguaaggacucagcugc | 1 | 0 | 1 | 0 | auuccaguaaggacucagcugcuaucaaacuacguuaagacuauaggaccaagguagugggaguggguuguaaacugca | 19:6586643..6586754:+ |
| gga-miR-N51 | uuaaucuagccugcucucuagu | 1 | 2 | 0 | 1 | augagagcagcucagauuagcccagacuaaagguuaaucuagccugcucucuagu | 7:3548306..3548416:- |
| gga-miR-N52 | accgggugcuguaggcuu | 1505 | 2093 | 1099 | 969 | ucgggccugguuaguacuuggauggggagaccuccugggaauaaccgggugcuguaggcuu | AADN03023593.1:487..595:+ |
| gga-miR-N53 | caggaacaacugaagugaauu | 48 | 91 | 82 | 88 | aaccaccucaguuguuucuacccuuucuguaaagcaggcaggaacaacugaagugaauu | 8:23481645..23481752:+ |

Note: z06, z14, z22, and z30 represent small the RNA libraries obtained using samples from chickens aged 6, 14, 22, and 30 weeks, respectively. The novel miRNAs were named using the “miR” prefix and a unique identifying number described by Ambros et al.(2003). In the temporary name, the “gga” prefix is abbreviation for *Gallus gallus*, and the “N” letter is short for novel.
