# Supplementary material for: MicroRNAs and their regulatory networks in Chinese Gushi chicken abdominal adipose tissue during postnatal late development
Source: BMC Genomics. 2019 Oct 25;20:778. doi: 10.1186/s12864-019-6094-2 (PMC6815035; doi:10.1186/s12864-019-6094-2)
Supplement: Supplementary file 9 — Additional file 9: Table S3. Details of the significant differentially expressed miRNAs identified in this study. [file 12864_2019_6094_MOESM9_ESM.docx]

**Table S3** Details of the significant differentially expressed miRNAs identified in this study

| miRNA ID | z14vsz06 | | | z22vsz06 | | | z22vsz14 | | | z30vsz06 | | | z30vsz14 | | | z30vsz22 | | |
| --- | --- | --- | --- | --- | --- | --- | --- | --- | --- | --- | --- | --- | --- | --- | --- | --- | --- | --- |
|  | log2.Fold_change. | q-value | Signifi | log2.Fold_change. | q-value | Signifi | log2.Fold_change | q-value | Signifi | log2.Fold_change | q-value | Signifi | log2.Fold_change | q-value | Signifi | log2.Fold_change | q-value | Signifi |
| gga-let-7j-3p | 1.1483 | 0.010272 | FALSE | 1.0829 | 3.91E-05 | TRUE | 0.20019 | 0.52623 | FALSE | 1.1059 | 9.54E-08 | TRUE | 0.16909 | 0.030544 | FALSE | 0.020531 | 0.61498 | FALSE |
| gga-miR-10a-5p | 1.1483 | 0 | TRUE | 1.0331 | 0 | TRUE | -0.06518 | 0.048007 | FALSE | 1.3212 | 0 | TRUE | 0.16881 | 0 | FALSE | 0.28562 | 0 | FALSE |
| gga-miR-122-3p | -1.8832 | 0.012418 | FALSE | -0.7165 | 0.43022 | FALSE | 1.2167 | 0.43033 | FALSE | -2.6893 | 0.003673 | TRUE | -0.81017 | 0.98262 | FALSE | -1.9753 | 0.15246 | FALSE |
| gga-miR-122-5p | -1.3169 | 3.46E-237 | TRUE | 0.0019714 | 0.094092 | FALSE | 1.3689 | ####### | TRUE | -1.9252 | ####### | TRUE | -0.61236 | 7.78E-05 | FALSE | -1.9296 | ####### | TRUE |
| gga-miR-125b-5p | 0.98195 | 1.38E-150 | FALSE | 0.42071 | 1.50E-26 | FALSE | -0.51116 | 4.47E-52 | FALSE | 1.0677 | 0 | TRUE | 0.081673 | 1.54E-69 | FALSE | 0.64446 | ####### | FALSE |
| gga-miR-130b-3p | 0.31761 | 0.971 | FALSE | -0.27761 | 0.16712 | FALSE | -0.54515 | 0.076969 | FALSE | -1.1961 | 1.79E-06 | TRUE | -1.5177 | 7.74E-08 | TRUE | -0.92098 | 0.005773 | FALSE |
| gga-miR-133a-3p | -5.4846 | 0 | TRUE | -5.2352 | 0 | TRUE | 0.29944 | 0.074082 | FALSE | -6.4579 | 0 | TRUE | -0.97728 | 0.002618 | FALSE | -1.2251 | 2.42E-08 | TRUE |
| gga-miR-133a-5p | -6.3959 | 3.78E-70 | TRUE | -5.6329 | 2.83E-71 | TRUE | 0.81314 | 0.972 | FALSE | -6.7247 | 5.57E-70 | TRUE | -0.33276 | 0.99742 | FALSE | -1.0943 | 0.76638 | FALSE |
| gga-miR-133b | -6.2076 | 8.52E-75 | TRUE | -5.7158 | 2.03E-75 | TRUE | 0.54183 | 0.99958 | FALSE | -6.8951 | 1.83E-73 | TRUE | -0.69152 | 0.98262 | FALSE | -1.1817 | 0.75348 | FALSE |
| gga-miR-133c-3p | -5.4597 | 1.08E-248 | TRUE | -5.2654 | 9.40E-247 | TRUE | 0.24436 | 0.99958 | FALSE | -6.7426 | ####### | TRUE | -1.2869 | 0.23933 | FALSE | -1.4797 | 0.040141 | FALSE |
| gga-miR-146c-3p | 0.90501 | 0.06583 | FALSE | 0.53348 | 0.37206 | FALSE | -0.32146 | 0.99958 | FALSE | 1.0111 | 0.000129 | TRUE | 0.1021 | 0.21135 | FALSE | 0.47518 | 0.026019 | FALSE |
| gga-miR-155 | 1.1741 | 1.42E-06 | TRUE | 0.57206 | 0.05077 | FALSE | -0.55195 | 0.040475 | FALSE | 0.86762 | 6.05E-07 | FALSE | -0.31049 | 0.98262 | FALSE | 0.29308 | 0.015257 | FALSE |
| gga-miR-1684a-3p | -0.22185 | 0.73674 | FALSE | -0.89185 | 0.17809 | FALSE | -0.61992 | 0.94511 | FALSE | 0.73451 | 0.086156 | FALSE | 0.95233 | 0.006067 | FALSE | 1.6239 | 0.000249 | TRUE |
| gga-miR-1729-5p | -1.7035 | 4.82E-13 | TRUE | -0.93421 | 2.10E-05 | FALSE | 0.81937 | 0.020492 | FALSE | -1.1754 | 8.59E-06 | TRUE | 0.52412 | 0.021428 | FALSE | -0.24362 | 0.99406 | FALSE |
| gga-miR-1744-3p | -4.8315 | 8.87E-05 | TRUE | -2.2694 | 0.005927 | TRUE | 2.6122 | 0.48059 | FALSE | -0.03924 | 0.96958 | FALSE | 4.7883 | 4.65E-05 | TRUE | 2.2277 | 0.00406 | TRUE |
| gga-miR-184-3p | 0.65908 | 0.31485 | FALSE | 1.0605 | 0.000387 | TRUE | 0.45149 | 0.10037 | FALSE | 0.54218 | 0.047907 | FALSE | -0.12094 | 0.84349 | FALSE | -0.5208 | 0.39107 | FALSE |
| gga-miR-187-3p | 2.2011 | 0.000848 | TRUE | 1.3756 | 0.098604 | FALSE | -0.77541 | 0.42944 | FALSE | 2.4222 | 8.06E-07 | TRUE | 0.21707 | 0.32344 | FALSE | 1.0441 | 0.005763 | TRUE |
| gga-miR-194 | 5.6119 | 1.38E-296 | TRUE | -0.14748 | 0.91249 | FALSE | -5.7093 | ####### | TRUE | -1.1043 | 0.086156 | FALSE | -6.7203 | ####### | TRUE | -0.95934 | 0.28244 | FALSE |
| gga-miR-196-5p | -0.21881 | 0.45655 | FALSE | 0.42021 | 0.55143 | FALSE | 0.68909 | 0.057393 | FALSE | 1.2528 | 3.48E-08 | TRUE | 1.4676 | 4.36E-12 | TRUE | 0.83013 | 1.89E-05 | FALSE |
| gga-miR-1a-3p | -4.8103 | 0 | TRUE | -4.205 | 0 | TRUE | 0.65534 | ####### | FALSE | -5.7533 | 0 | TRUE | -0.94702 | 1.45E-83 | FALSE | -1.5507 | 0 | TRUE |
| gga-miR-1b-3p | -1.8487 | 4.41E-122 | TRUE | -3.6378 | 6.82E-221 | TRUE | -1.739 | 1.33E-21 | TRUE | -3.7179 | ####### | TRUE | -1.8733 | 5.49E-20 | TRUE | -0.0826 | 0.99033 | FALSE |
| gga-miR-200a-3p | 4.7271 | 0 | TRUE | 4.8134 | 0 | TRUE | 0.13641 | 5.62E-08 | FALSE | -0.23081 | 0.90033 | FALSE | -4.9619 | 0 | TRUE | -5.0467 | 0 | TRUE |
| gga-miR-200a-5p | 4.7553 | 6.31E-19 | TRUE | 4.2792 | 1.39E-14 | TRUE | -0.42601 | 0.58218 | FALSE | 0.002582 | 0.96958 | FALSE | -4.7568 | 9.47E-19 | TRUE | -4.2791 | 2.38E-14 | TRUE |
| gga-miR-200b-3p | 4.769 | 0 | TRUE | 4.2723 | 0 | TRUE | -0.44669 | 5.68E-15 | FALSE | 0.91153 | 6.58E-09 | FALSE | -3.8615 | 0 | TRUE | -3.3632 | 0 | TRUE |
| gga-miR-200b-5p | 4.0308 | 0.000151 | TRUE | 4.3904 | 1.45E-06 | TRUE | 0.40969 | 0.78555 | FALSE | -0.42626 | 0.96958 | FALSE | -4.4611 | 8.39E-05 | TRUE | -4.8192 | 9.93E-07 | TRUE |
| gga-miR-202-5p |  |  |  |  |  |  | 2.3492 | 0.89447 | FALSE | 6.3438 | 1.47E-10 | TRUE | 6.7293 | 2.04E-10 | TRUE | 4.4318 | 2.93E-10 | TRUE |
| gga-miR-203a | 1.5461 | 0.000114 | TRUE | 1.0176 | 0.020079 | FALSE | -0.47841 | 0.52638 | FALSE | -0.61959 | 0.57071 | FALSE | -2.1697 | 3.65E-07 | TRUE | -1.6397 | 0.000385 | TRUE |
| gga-miR-204 | 1.28 | 0.000229 | TRUE | 0.86476 | 0.016217 | FALSE | -0.36516 | 0.76606 | FALSE | 1.136 | 3.95E-06 | TRUE | -0.14805 | 0.83843 | FALSE | 0.26874 | 0.12074 | FALSE |
| gga-miR-206 | -7.6959 | 1.48E-129 | TRUE | -7.0431 | 1.36E-137 | TRUE | 0.70283 | 0.99958 | FALSE | -6.5536 | ####### | TRUE | 1.1382 | 0.40585 | FALSE | 0.487 | 0.84339 | FALSE |
| gga-miR-215-5p | 6.0261 | 0 | TRUE | 0.36448 | 0.094092 | FALSE | -5.6115 | 0 | TRUE | -0.74996 | 0.000114 | FALSE | -6.7801 | 0 | TRUE | -1.1169 | 9.90E-10 | TRUE |
| gga-miR-217-5p | 3.6077 | 6.05E-06 | TRUE | 1.905 | 0.14056 | FALSE | -1.6526 | 0.01443 | FALSE | -1.0112 | 0.90033 | FALSE | -4.6229 | 5.94E-07 | TRUE | -2.9187 | 0.040131 | FALSE |
| gga-miR-223 | -0.19973 | 0.097216 | FALSE | -0.54167 | 0.006752 | FALSE | -0.29187 | 0.94511 | FALSE | 0.76669 | 5.40E-08 | FALSE | 0.96239 | 1.23E-14 | FALSE | 1.3059 | 7.01E-18 | TRUE |
| gga-miR-29b-3p | 1.3608 | 0.019708 | FALSE | 1.6758 | 0.000107 | TRUE | 0.36513 | 0.52166 | FALSE | 1.9473 | 1.73E-08 | TRUE | 0.58253 | 0.004833 | FALSE | 0.26903 | 0.2401 | FALSE |
| gga-miR-31-5p | 2.5614 | 0.003872 | TRUE | 2.4622 | 0.002867 | TRUE | -0.04912 | 0.99958 | FALSE | 1.2847 | 0.28333 | FALSE | -1.2807 | 0.27634 | FALSE | -1.18 | 0.23301 | FALSE |
| gga-miR-34b-3p | 8.2447 | 2.15E-88 | TRUE | 7.5256 | 2.37E-62 | TRUE | -0.66902 | 1.47E-06 | FALSE | 0.42618 | 0.96958 | FALSE | -7.8225 | 7.16E-93 | TRUE | -7.1019 | 1.25E-64 | TRUE |
| gga-miR-34b-5p | 8.7427 | 0 | TRUE | 8.7619 | 0 | TRUE | 0.069343 | 0.000357 | FALSE | 1.2827 | 0.077614 | FALSE | -7.464 | 0 | TRUE | -7.4817 | 0 | TRUE |
| gga-miR-34c-3p | 8.3467 | 6.86E-32 | TRUE | 7.5999 | 2.71E-22 | TRUE | -0.69672 | 0.007877 | FALSE | 0.89567 | 0.96015 | FALSE | -7.455 | 1.13E-34 | TRUE | -6.7067 | 1.12E-23 | TRUE |
| gga-miR-34c-5p | 8.2239 | 3.02E-274 | TRUE | 8.1759 | 5.61E-289 | TRUE | 0.002073 | 0.40186 | FALSE | 0.3847 | 0.90033 | FALSE | -7.8432 | ####### | TRUE | -7.7937 | ####### | TRUE |
| gga-miR-3531-5p | -0.27025 | 0.66993 | FALSE | -0.014392 | 0.94907 | FALSE | 0.30594 | 0.99958 | FALSE | 0.94458 | 0.011427 | FALSE | 1.2108 | 0.000242 | TRUE | 0.95649 | 0.005773 | FALSE |
| gga-miR-365-3p | 0.60538 | 1.40E-05 | FALSE | 0.27558 | 0.073604 | FALSE | -0.27973 | 0.091365 | FALSE | 1.0939 | 3.14E-64 | TRUE | 0.48451 | 1.15E-34 | FALSE | 0.81587 | 1.44E-48 | FALSE |
| gga-miR-365b-5p | 0.55093 | 0.25445 | FALSE | 0.45279 | 0.14762 | FALSE | -0.04806 | 0.99958 | FALSE | 1.1496 | 3.86E-14 | TRUE | 0.59462 | 4.63E-09 | FALSE | 0.69431 | 2.42E-08 | FALSE |
| gga-miR-375 | 2.78 | 2.54E-26 | TRUE | 2.0494 | 4.61E-13 | TRUE | -0.68056 | 0.003469 | FALSE | -2.8783 | 1.83E-05 | TRUE | -5.6624 | 3.03E-43 | TRUE | -4.9302 | 5.08E-28 | TRUE |
| gga-miR-429-3p | 4.0425 | 2.54E-26 | TRUE | 4.2512 | 6.24E-35 | TRUE | 0.2588 | 0.20525 | FALSE | -0.34137 | 0.96958 | FALSE | -4.3879 | 2.35E-27 | TRUE | -4.595 | 5.92E-36 | TRUE |
| gga-miR-449a | 7.8202 | 4.22E-12 | TRUE | 7.3178 | 7.87E-10 | TRUE | -0.45235 | 0.76606 | FALSE |  |  |  | -6.8677 | 5.90E-13 | TRUE | -6.4348 | 3.09E-10 | TRUE |
| gga-miR-454-3p | 0.53545 | 0.25849 | FALSE | -0.17907 | 0.50051 | FALSE | -0.66445 | 0.017191 | FALSE | -1.3619 | 8.33E-07 | TRUE | -1.9014 | 2.02E-11 | TRUE | -1.1853 | 0.000373 | TRUE |
| gga-miR-460b-5p | 1.4539 | 0.27559 | FALSE | 2.3238 | 0.001221 | TRUE | 0.92002 | 0.20424 | FALSE | 0.42033 | 0.90033 | FALSE | -1.0376 | 0.74244 | FALSE | -1.906 | 0.009189 | TRUE |
| gga-miR-499-3p | -5.6342 | 4.45E-07 | TRUE | -5.7044 | 4.32E-07 | TRUE |  |  |  | -5.8004 | 4.58E-07 | TRUE |  |  |  |  |  |  |
| gga-miR-499-5p | -9.183 | 0 | TRUE | -6.8657 | 0 | TRUE | 2.3674 | 6.87E-21 | TRUE | -10.471 | 0 | TRUE | -1.2919 | 0.12911 | FALSE | -3.6077 | 4.78E-29 | TRUE |
| gga-miR-9-5p | 1.3086 | 2.54E-11 | TRUE | 1.4106 | 6.51E-18 | TRUE | 0.15211 | 0.24247 | FALSE | 1.5066 | 8.50E-29 | TRUE | 0.19392 | 3.49E-05 | FALSE | 0.093429 | 0.042231 | FALSE |
| gga-miR-99a-5p | 1.1684 | 0 | TRUE | 0.81905 | 0 | FALSE | -0.29927 | 1.65E-72 | FALSE | 0.66367 | 0 | FALSE | -0.50876 | 9.18E-51 | FALSE | -0.15786 | 0.008364 | FALSE |
| novel_56 | -0.65528 | 8.07E-13 | FALSE | -0.87051 | 1.39E-14 | FALSE | -0.16515 | 0.99958 | FALSE | -1.3245 | 3.01E-21 | TRUE | -0.67324 | 0.07835 | FALSE | -0.45646 | 0.2401 | FALSE |

Note: z06, z14, z22, and z30 represent small the RNA libraries obtained using samples from chickens aged 6, 14, 22, and 30 weeks, respectively.
