# Supplementary material for: MicroRNAs and their regulatory networks in Chinese Gushi chicken abdominal adipose tissue during postnatal late development
Source: BMC Genomics. 2019 Oct 25;20:778. doi: 10.1186/s12864-019-6094-2 (PMC6815035; doi:10.1186/s12864-019-6094-2)
Supplement: Supplementary file 10 — Additional file 10: Table S4. The main biological processes associated with lipid metabolism or fat deposition during abdominal adipose tissue development in Gushi chicken. [file 12864_2019_6094_MOESM10_ESM.docx]

**Table S4** The main biological processes associated with lipid metabolism or fat deposition during abdominal adipose tissue development in Gushi chicken

| GO accession | Description | z14 *vs* z06 | | z22 *vs* z14 | | z30 *vs* z22 | |
| --- | --- | --- | --- | --- | --- | --- | --- |
|  |  | Corrected p-value | DEG item | Corrected p-value | DEG item | Corrected p-value | DEG item |
| GO:0045444 | fat cell differentiation | 0.75649 | 40 | 1 | 8 | 0.70181 | 37 |
| GO:0045598 | regulation of fat cell differentiation | 0.84106 | 22 | 1 | 3 | 0.70858 | 21 |
| GO:0045599 | negative regulation of fat cell differentiation | 0.76952 | 10 | 1 | 1 | 0.87073 | 9 |
| GO:0070345 | negative regulation of fat cell proliferation | 1 | 1 | 1 | 1 | 1 | 1 |
| GO:0045600 | positive regulation of fat cell differentiation | 1 | 10 | 1 | 1 | 1 | 10 |
| GO:0008610 | lipid biosynthetic process | 0.15394 | 101 | 0.14074 | 43 | 0.20351 | 91 |
| GO:0016042 | lipid catabolic process | 0.97128 | 35 | 1 | 10 | 1 | 28 |
| GO:0030259 | lipid glycosylation | 0.84106 | 3 | 0.97011 | 2 | 0.78072 | 3 |
| GO:0055088 | lipid homeostasis | 1 | 15 | 1 | 3 | 1 | 14 |
| GO:0010876 | lipid localization | 1 | 38 | 1 | 16 | 1 | 34 |
| GO:0006629 | lipid metabolic process | 0.13181 | 208 | 0.19606 | 80 | 0.12025 | 189 |
| GO:0030258 | lipid modification | 0.59135 | 33 | 1 | 11 | 0.87073 | 28 |
| GO:0034440 | lipid oxidation | 0.34643 | 16 | 1 | 3 | 0.67077 | 13 |
| GO:0034389 | lipid particle organization | 1 | 3 | 1 | 1 | 1 | 3 |
| GO:0046834 | lipid phosphorylation | 1 | 8 | 1 | 4 | 1 | 7 |
| GO:0019915 | lipid storage | 1 | 7 | 1 | 1 | 1 | 4 |
| GO:0034204 | lipid translocation | 1 | 3 | 1 | 2 | 1 | 3 |
| GO:0006869 | lipid transport | 1 | 35 | 1 | 16 | 1 | 33 |
| GO:0046890 | regulation of lipid biosynthetic process | 0.68218 | 23 | 1 | 9 | 0.43523 | 23 |
| GO:0050994 | regulation of lipid catabolic process | 0.87553 | 7 | 1 | 1 | 0.87073 | 7 |
| GO:0043550 | regulation of lipid kinase activity | 0.87553 | 11 | 1 | 1 | 0.87073 | 11 |
| GO:0019216 | regulation of lipid metabolic process | 0.73674 | 46 | 1 | 11 | 0.28774 | 47 |
| GO:0010883 | regulation of lipid storage | 1 | 5 | 1 | 1 | 1 | 3 |
| GO:0032368 | regulation of lipid transport | 1 | 12 | 1 | 5 | 1 | 10 |
| GO:0051055 | negative regulation of lipid biosynthetic process | 0.68218 | 12 | 1 | 5 | 0.51462 | 12 |
| GO:0045833 | negative regulation of lipid metabolic process | 0.33975 | 17 | 1 | 5 | 0.17818 | 17 |
| GO:0010888 | negative regulation of lipid storage | 0.87553 | 4 | 1 | 1 | 1 | 3 |
| GO:0032369 | negative regulation of lipid transport | 1 | 3 | 1 | 1 | 1 | 3 |
| GO:0046889 | positive regulation of lipid biosynthetic process | 0.87553 | 8 | 1 | 2 | 1 | 7 |
| GO:0045834 | positive regulation of lipid metabolic process | 0.93653 | 17 | 1 | 2 | 0.87073 | 16 |
| GO:0032370 | positive regulation of lipid transport | 0.87553 | 9 | 1 | 3 | 1 | 7 |
| GO:0044242 | cellular lipid catabolic process | 0.87553 | 26 | 1 | 9 | 1 | 21 |
| GO:0044255 | cellular lipid metabolic process | 0.42615 | 149 | 0.767 | 56 | 0.59219 | 132 |
| GO:0006635 | fatty acid beta-oxidation | 0.67056 | 10 | 1 | 3 | 0.87073 | 8 |
| GO:0033539 | fatty acid beta-oxidation using acyl-CoA dehydrogenase | 1 | 1 | 1 | 1 | 1 | 1 |
| GO:0006633 | fatty acid biosynthetic process | 0.29568 | 26 | 0.091249 | 15 | 0.58977 | 22 |
| GO:0009062 | fatty acid catabolic process | 0.24046 | 15 | 1 | 5 | 0.62689 | 12 |
| GO:1901570 | fatty acid derivative biosynthetic process | 1 | 4 | 1 | 2 | 1 | 4 |
| GO:1901568 | fatty acid derivative metabolic process | 1 | 9 | 1 | 4 | 0.99742 | 11 |
| GO:1901571 | fatty acid derivative transport | 1 | 5 | 1 | 3 | 1 | 5 |
| GO:0030497 | fatty acid elongation | 0.67056 | 5 | 0.97011 | 3 | 0.54959 | 5 |
| GO:0019367 | fatty acid elongation, saturated fatty acid | 0.87553 | 2 | 1 | 1 | 0.87073 | 2 |
| GO:0055089 | fatty acid homeostasis | 1 | 2 | 1 | 1 | 1 | 2 |
| GO:0019395 | fatty acid oxidation | 0.39188 | 15 | 1 | 3 | 0.58977 | 13 |
| GO:1902001 | fatty acid transmembrane transport | 0.87553 | 2 | 1 | 1 | 0.87073 | 2 |
| GO:0015908 | fatty acid transport | 0.87553 | 11 | 1 | 5 | 1 | 9 |
| GO:0042304 | regulation of fatty acid biosynthetic process | 0.87553 | 5 | 0.97011 | 3 | 0.87073 | 5 |
| GO:0019217 | regulation of fatty acid metabolic process | 0.68218 | 12 | 1 | 3 | 0.51462 | 12 |
| GO:2000191 | regulation of fatty acid transport | 0.85608 | 5 | 0.97011 | 3 | 0.78072 | 5 |
| GO:0045717 | negative regulation of fatty acid biosynthetic process | 0.99078 | 3 | 1 | 2 | 0.94027 | 3 |
| GO:0045922 | negative regulation of fatty acid metabolic process | 0.87553 | 4 | 1 | 2 | 0.87073 | 4 |
| GO:2000192 | negative regulation of fatty acid transport | 0.84106 | 3 | 1 | 1 | 0.78072 | 3 |
| GO:0045723 | positive regulation of fatty acid biosynthetic process | 1 | 1 | 1 | 1 | 1 | 1 |
| GO:0045923 | positive regulation of fatty acid metabolic process | 1 | 3 | 1 | 1 | 1 | 3 |
| GO:2000193 | positive regulation of fatty acid transport | 1 | 2 | 1 | 1 | 1 | 2 |
| GO:0001676 | long-chain fatty acid metabolic process | 1 | 6 | 1 | 3 | 1 | 7 |
| GO:0042759 | long-chain fatty acid biosynthetic process | 0.84106 | 3 | 1 | 1 | 1 | 2 |
| GO:0044539 | long-chain fatty acid import | 1 | 1 | 1 | 1 | 1 | 1 |
| GO:0015909 | long-chain fatty acid transport | 0.80119 | 9 | 0.97011 | 5 | 0.87073 | 8 |
| GO:0035336 | long-chain fatty-acyl-CoA metabolic process | 0.87553 | 2 | 1 | 1 | 1 | 1 |
| GO:0042761 | very long-chain fatty acid biosynthetic process | 0.70096 | 4 | 1 | 2 | 0.62689 | 4 |
| GO:0000038 | very long-chain fatty acid metabolic process | 0.84106 | 7 | 1 | 3 | 0.69789 | 7 |
| GO:0006636 | unsaturated fatty acid biosynthetic process | 1 | 6 | 1 | 3 | 1 | 4 |
| GO:0033559 | unsaturated fatty acid metabolic process | 1 | 11 | 1 | 5 | 1 | 11 |
| GO:0006695 | cholesterol biosynthetic process | 0.97128 | 5 | 1 | 3 | 0.87073 | 5 |
| GO:0006707 | cholesterol catabolic process | 0.87553 | 2 | 1 | 1 | 1 | 1 |
| GO:0033344 | cholesterol efflux | 1 | 5 | 1 | 1 | 1 | 4 |
| GO:0034435 | cholesterol esterification | 1 | 1 | 1 | 1 | 1 | 1 |
| GO:0042632 | cholesterol homeostasis | 1 | 11 | 1 | 2 | 1 | 11 |
| GO:0008203 | cholesterol metabolic process | 0.87553 | 14 | 0.74216 | 9 | 0.87073 | 14 |
| GO:0030301 | cholesterol transport | 1 | 6 | 1 | 2 | 1 | 5 |
| GO:0045541 | negative regulation of cholesterol biosynthetic process | 1 | 1 | 0.87073 | 2 | 1 | 1 |
| GO:0090205 | positive regulation of cholesterol metabolic process | 1 | 1 | 1 | 1 | 0.87073 | 2 |
| GO:0008654 | phospholipid biosynthetic process | 0.56868 | 27 | 0.91878 | 12 | 0.87073 | 22 |
| GO:0009395 | phospholipid catabolic process | 1 | 7 | 1 | 2 | 1 | 6 |
| GO:0046839 | phospholipid dephosphorylation | 1 | 5 | 1 | 1 | 1 | 4 |
| GO:0006644 | phospholipid metabolic process | 0.84615 | 58 | 1 | 21 | 0.87073 | 50 |
| GO:0045332 | phospholipid translocation | 1 | 2 | 1 | 2 | 1 | 2 |
| GO:0015914 | phospholipid transport | 1 | 8 | 1 | 5 | 1 | 9 |

[Note:](javascript:void(0);) z06, z14, z22, and z30 represent small the RNA libraries obtained using samples from chickens aged 6, 14, 22, and 30 weeks, respectively.
